# Electronic perturbation of Cu nanowire surfaces with functionalized graphdiyne for enhanced CO_2_ reduction reaction

**DOI:** 10.1093/nsr/nwae253

**Published:** 2024-07-25

**Authors:** Haiyuan Zou, Dongfang Cheng, Chao Tang, Wen Luo, Huatian Xiong, Hongliang Dong, Fan Li, Tao Song, Siyan Shu, Hao Dai, Ziang Cui, Zhouguang Lu, Lele Duan

**Affiliations:** Department of Chemistry, Southern University of Science and Technology, Shenzhen 518055, China; Department of Chemical and Biomolecular Engineering, University of California Los Angeles, Los Angeles 90095, USA; Department of Chemical and Biomolecular Engineering, University of California Los Angeles, Los Angeles 90095, USA; Department of Chemistry, Southern University of Science and Technology, Shenzhen 518055, China; Department of Materials Science and Engineering, Southern University of Science and Technology, Shenzhen 518055, China; Center of Artificial Photosynthesis for Solar Fuels and Department of Chemistry, School of Science, Westlake University, Hangzhou 310030, China; Center for High-Pressure Science and Technology Advanced Research, Shanghai 201203, China; Department of Chemistry, Southern University of Science and Technology, Shenzhen 518055, China; Department of Chemistry, Southern University of Science and Technology, Shenzhen 518055, China; Department of Chemistry, Southern University of Science and Technology, Shenzhen 518055, China; Department of Chemistry, Southern University of Science and Technology, Shenzhen 518055, China; Department of Chemistry, Southern University of Science and Technology, Shenzhen 518055, China; Department of Chemistry, Tsinghua University, Beijing 100084, China; Department of Materials Science and Engineering, Southern University of Science and Technology, Shenzhen 518055, China; Department of Chemistry, Southern University of Science and Technology, Shenzhen 518055, China; Center of Artificial Photosynthesis for Solar Fuels and Department of Chemistry, School of Science, Westlake University, Hangzhou 310030, China; Division of Solar Energy Conversion and Catalysis at Westlake University, Zhejiang Baima Lake Laboratory Co., Ltd, Hangzhou 310000, China; Institute of Natural Sciences, Westlake Institute for Advanced Study, Hangzhou 310024, China

**Keywords:** CO_2_ reduction reaction, copper nanowire, group-functionalized graphdiyne, surface electronic perturbation

## Abstract

Electronic perturbation of the surfaces of Cu catalysts is crucial for optimizing electrochemical CO_2_ reduction activity, yet still poses great challenges. Herein, nanostructured Cu nanowires (NW) with fine-tuned surface electronic structure are achieved via surface encapsulation with electron-withdrawing (–F) and -donating (–Me) group-functionalized graphdiynes (R-GDY, R = –F and –Me) and the resulting catalysts, denoted as R-GDY/Cu NW, display distinct CO_2_ reduction performances. *In situ* electrochemical spectroscopy revealed that the *CO (a key intermediate of the CO_2_ reduction reaction) binding affinity and consequent *CO coverage positively correlate with the Cu surface oxidation state, leading to favorable C–C coupling on F-GDY/Cu NW over Me-GDY/Cu NW. Electrochemical measurements corroborate the favorable C_2_H_4_ production with an optimum C_2+_ selectivity of 73.15% ± 2.5% observed for F-GDY/Cu NW, while the predominant CH_4_ production is favored by Me-GDY/Cu NW. Furthermore, by leveraging the *Cu–hydroxyl (OH)/*CO ratio as a descriptor, mechanistic investigation reveals that the protonation of distinct adsorbed *CO facilitated by *Cu–OH is crucial for the selective generation of C_2_H_4_ and CH_4_ on F-GDY/Cu NW and Me-GDY/Cu NW, respectively.

## Teaser

This paper introduces Cu nanowires encapsulated with functionalized graphdiynes, which catalyze electrochemical CO2 reduction selectively to C2H4 or CH4 depending on the electronic structures of surface Cu atoms perturbated by graphdiynes.

## INTRODUCTION

Electrochemical conversion of CO_2_ into value-added chemical feedstock and fuels represents a promising avenue for both renewable energy storage and carbon emissions mitigation [1–3]. Among the explored catalysts, copper (Cu) emerged as a well-known model catalyst capable of reducing CO_2_ into hydrocarbons and/or oxygenates, owing to its judiciously balanced chemisorption energy of intermediates [4–6]. Nevertheless, the electrochemical conversion process necessitates multiple proton and electron transfers, posing a substantial challenge for Cu catalysts with constrained product selectivity.

Given that the protonation or dimerization of adsorbed CO (*CO) on the Cu surface has been identified as a rate-determining step towards C_1_ or C_2+_ products, the catalyst surface properties thus play a critical role in steering the pathway of the electrochemical CO_2_ reduction reaction (CO_2_RR) [7–12]. Particularly, the electronic perturbation of the copper surface governs a potent approach to fine-tune intermediate adsorption, thereby aiding in circumventing the sluggish kinetic bottleneck of the CO_2_RR [13–16]. For example, research has showcased that the N−C layer on the Cu surface, with its confinement effect and electron-donating capabilities, efficiently suppresses the deoxygenation of the intermediate HOCCH* to ethylene and promotes ethanol production [17]. Modification of Cu surfaces with polyaromatic films was found to boost the selectivity towards C_2+_ products [18,19]. Moreover, recent breakthroughs have suggested that the chelating N-heterocyclic carbene (NHC) ligands bound to a Pd catalyst can engender an electron-rich Pd surface through the robust σ-donation from the NHC ligands to the Pd, thus facilitating the HCOO^−^ formation [20]. The incumbent strategies, however, still suffer limited freedom to precisely perturbate the Cu surface without blocking the active centers on those carbonaceous-modified Cu surfaces [21]. Furthermore, the primary concerns regarding the low stability and conductivity inherent in molecular additives constrain their practical implementation, particularly in scenarios with high current density required [22–24]. Therefore, seeking a suitable surface modulator that mutually combines the merits of both carbon materials and molecular additives is highly desired but remains underexplored.

Graphdiyne (GDY)—an emerging allotrope of carbon—in addition to its derivatives, features tunable topology and electronic structure with excellent conductivity [25,26]. Specifically, by exquisitely tailoring the chemical structure of the GDY monomer with electron-withdrawing/-donating groups, tens of GDY derivatives have been prepared with precisely perturbated electronic properties [27,28]. In this scenario, incorporating group-functionalized GDY derivatives onto the Cu surface is envisioned to regulate the surface electronic properties of Cu in a controlled fashion. Moreover, the ordered pores formed by butadiyne linkages (–C≡C–C≡C–) between benzene rings within the GDY derivatives guarantee active site exposure, and enable mass and product transportation, making it an ideal platform for Cu surface modulation [29].

**Figure 1.**
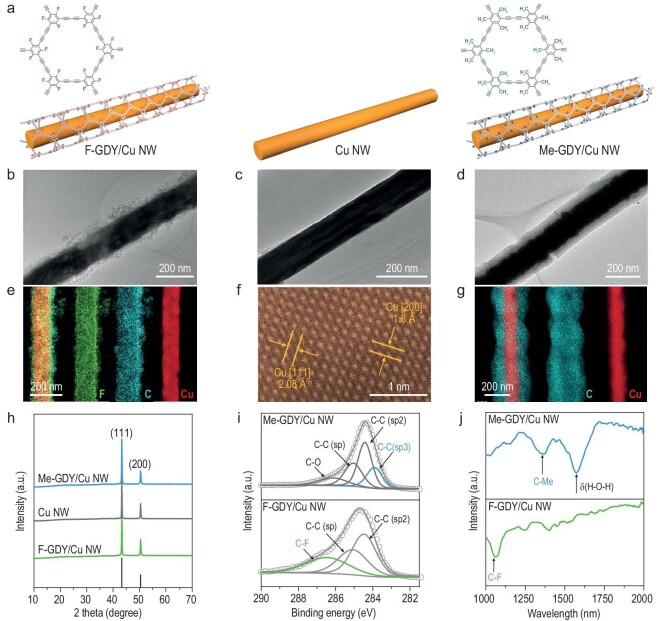
Morphologies and structural characterizations. (a) Schematic illustration of catalyst composites. (b–d) TEM images of Cu NW and R-GDY/Cu NW. EDS images of (e) F-GDY/Cu NW and (g) Me-GDY/Cu NW. (f) HAADF-STEM image of Cu NW. (h) XRD patterns of Cu NW and R-GDY/Cu NW. (i) C 1s XPS and (j) FTIR spectra of R-GDY/Cu NW.

Herein, we elaborately engineered the surface of Cu nanowires with GDY derivatives (the resulting catalysts are denoted as R-GDY/Cu NW; R = –F and –Me; Fig. 1a) by using *in situ* cross-coupling of GDY monomers that were functionalized with electron-withdrawing and -donating groups (–F and –Me), and further explored their structure–function relationships in the CO_2_RR. Comprehensive analyses revealed a precise electronic perturbation of the Cu NW surface by leveraging electron-withdrawing and -donating group-functionalized R-GDY, leading to a higher Cu oxidation state of F-GDY/Cu NW than Me-GDY/Cu NW. *In situ* spectroscopy indicated that the binding strength of *CO is proportionally determined by the Cu surface oxidation state of R-GDY/Cu NW, imparting an enriched *CO coverage and a more favorable C–C coupling process on F-GDY/Cu NW compared with Me-GDY/Cu NW. Consequently, F-GDY/Cu NW achieved a preferential C_2_H_4_ production with an optimal selectivity of 73.15% ± 2.5% for C_2+_ products, while Me-GDY/Cu NW predominantly favors C_1_ products, particularly CH_4_ generation. Furthermore, by introducing the ratio of *Cu–hydroxyl (OH)/*CO as a valuable descriptor, mechanistic insights unveil that the protonation of distinctly adsorbed *CO mediated by *Cu–OH is pivotal for the preferential formation of C_2_H_4_ and CH_4_ on F-GDY/Cu NW and Me-GDY/Cu NW, respectively. This work offers valuable insights into the controlled perturbation of the electronic structure of Cu nanocatalyst surfaces, holding promise for future endeavors in refining catalysts for the CO_2_RR.

## RESULTS AND DISCUSSION

Firstly, the meta-substituted GDY monomers with different electron-withdrawing/-donating groups of –F and –Me were synthesized according to the literature methods (see their synthesis procedures in Scheme S1) and their ^1^H nuclear magnetic resonance (^1^H NMR) spectra (Figs S1 and S2) are in line with the documented data [30–32]. Then, Cu nanowires were synthesized using a previously established method [33]. Scanning electron microscopy (SEM) and transmission electron microscopy (TEM) images showed that the as-prepared Cu NW exhibited ultra-long nanowire morphologies with uniform diameters ranging from 140 to 180 nm (Fig. 1c and Fig. S3a). The aberration-corrected high-angle annular dark-field scanning transmission electron microscopy (HAADF-STEM) image revealed the characteristic spacing of 2.08 and 1.81 Å, corresponding to the (111) and (200) lattice planes of face-centered cubic copper (Fig. 1f). To realize electronic perturbation at the Cu NW interface, an appropriate amount of functional group-tailored GDY derivatives were *in situ* grown on the Cu NW surface through the cross-coupling reaction of their corresponding monomers, leading to the resultant F-GDY/Cu NW and Me-GDY/Cu NW catalysts (Fig. 1a). SEM and TEM images clearly revealed a core-shell-like structure, in which the Cu NW surface was evenly coated with a carbon layer comprising F-GDY or Me-GDY (Fig. S3b and c, and Fig. 1b and d). In addition, energy-dispersive X-ray spectroscopy (EDS) analysis in STEM mode demonstrated the distribution of Cu signal along the NW, encapsulated by C, F elements in F-GDY/Cu NW and C element in the Me-GDY/Cu NW, respectively (Fig. 1e and g). A higher Brunauer–Emmett–Teller surface area was also achieved by the R-GDY/Cu NW samples compared with the bare Cu NW (Table S1).

Furthermore, structure and phase characteristics were analysed using powder X-ray diffraction (XRD) and X-ray photoelectron spectra (XPS). Figure 1h displays analogous XRD profiles of the as-prepared samples, with characteristic peaks at 23^o^ and 46^o^ matching the indexed cubic structure of copper (PDF No. 85-1326), which is consistent with the aforementioned TEM results. As shown in Fig. 1i, deconvolution of C 1s core-level spectra revealed characteristic C≡C (*sp*) and C = C (*sp2*) peaks for both samples. Notably, the characteristic C–F peak was exclusively observed in F-GDY/Cu NW while the C–C (*sp*^3^) peak was predominantly detected in Me-GDY/Cu NW. Simultaneously, Fourier-transform infrared spectroscopy (FTIR) spectra exhibited fingerprint vibration bands of C–F and C–CH_3_ on F-GDY/Cu NW and Me-GDY/Cu NW, respectively (Fig. 1j). In contrast, no characteristic FTIR bands of R-GDY were observed on the bare Cu NW (Fig. S4). Another band at 1645 cm^–1^ was assigned to the bending vibration of H_2_O molecules adsorbed on the surface [34]. Taken together, the above analysis unambiguously showcases the successful construction of functionalized R-GDY-coated Cu NW.

**Figure 2.**
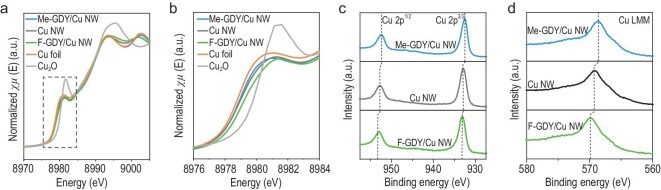
Electronic structure analysis. (a) XANES spectra and (b) enlarged near-edge position of as-prepared samples with reference Cu foil and Cu_2_O at Cu *K*-edge. (c) XPS spectra of Cu 2p and (d) Au spectra of Cu LMM for the as-prepared samples.

Synchrotron-based X-ray absorption spectroscopy was then employed to identify the electronic and local coordination structures. Figure 2a illustrates the X-ray absorption near-edge structure (XANES) at the Cu *K*-edge, comparing the prepared samples with the reference samples. The XANES profiles of the prepared samples closely resembled Cu foil, indicating their metallic Cu nature. Remarkably, close comparison of the near-edge positions of Cu *K*-edge XANES revealed a positively shifted energy trend from Me-GDY/Cu NW to F-GDY/Cu NW (Fig. 2b). A similar increase trend was also reflected in the corresponding first-order derivatives of Cu *K*-edge XANES (Fig. S5), suggesting the elevated average valance state of Cu as the electron-withdrawing ability of functionalized R-GDY increased. The Cu 2p XPS spectra in Fig. 2c further confirm the interface electronic perturbations in Cu^δ+^ states. Notably, the Cu 2p^3/2^ and Cu 2p^1/2^ spin–orbit peaks consistently shift to lower energy levels from F-GDY/Cu NW to Cu NW, and further to Me-GDY/Cu NW. Auger electron spectroscopy of Cu LMM further illustrated the distinct Cu valence state by leveraging the electron-withdrawing/-donating ability of R-GDY (Fig. 2d). Collectively, the above findings unambiguously evidenced the capacity to fine-tune the interface electronic structure of Cu NW through precisely tailoring the electron-withdrawing/-donating ability of surface-functionalized R-GDY.

Additionally, the Fourier-transformed *k*^2^-weighted extended X-ray absorption fine structure at the Cu *K*-edge revealed a prominent Cu–Cu peak at 2.2 Å within the as-prepared samples, and no discernible peaks corresponding to Cu–F or Cu–C bonds could be observed (Fig. S6). This implies that R-GDY does not chelate with surface Cu atoms, signifying that the surface decoration solely induces a distinct electronic structure of Cu without altering its coordination structure. Hence, the electron-donating and -withdrawing groups-functionalized R-GDY plays the role of ‘surface charge-state modulator’, enabling the exquisite electronic perturbation of Cu NW interfaces.

**Figure 3.**
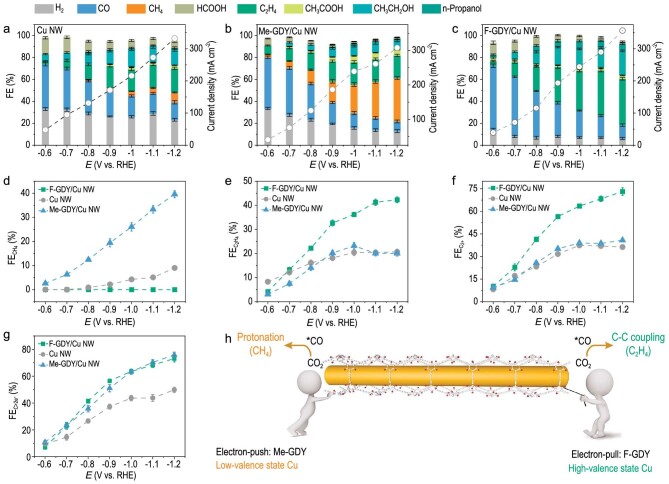
Electrochemical CO_2_RR performance. FE of production distribution of (a) Cu NW, (b) Me-GDY/Cu NW and (c) F-GDY/Cu NW in 1.0 M KOH electrolyte. Comparison of (d) $F{{E}_{{\mathrm{C}}{{{\mathrm{H}}}_4}}}$, (e) $F{{E}_{{{{\mathrm{C}}}_2}{{{\mathrm{H}}}_4}}}$, (f) $FE_{{\rm C}_{2^ +}}$ and (g)${\mathrm{\ }}F{{E}_{{\mathrm{C}} > 2{{e}^ - }}}$. (h) Schematic illustration of electron-push/pull by R-GDY on Cu NW surface for different product formation.

We then sought to identify the trade-off between the distinct interfacial electronic properties of Cu NW and CO_2_RR performance using a three-compartment flow electrolyser with a 1.0 M KOH solution as the electrolyte. Gas- and liquid-phase products were detected and quantified by using gas chromatography and NMR spectroscopy, respectively. Firstly, linear sweep voltammetry (LSV) of as-prepared samples was evaluated under CO_2_- and Ar-purged conditions. Figure S7 illustrates a significant current density gap between CO_2_- and Ar-purged LSV curves, suggesting the substantial contribution of the CO_2_RR on the catalyst surface for the higher current density delivered. Then, a wide potential range spanning from −0.6 to −1.2 V (versus reversible hydrogen electrode, RHE) was applied to investigate the catalytic behavior. Of note, all potentials in this work are referenced to as RHE unless otherwise specified. Figure 3a–c show the potential-dependent Faradaic efficiencies (*FE*s) of different products obtained using bare Cu NW, F-GDY/Cu NW and Me-GDY/Cu NW catalysts. For the bare Cu NW, the main CO_2_RR products are CO, CH_4_, C_2_H_4_, acetate, ethanol and n-propanol. The *FE* of CO (*FE*_CO_) reached a maximum of 40.8% at the beginning potential of –0.6 V, which decreased stepwise as the potential negatively shifted. In contrast, the *FE* of the more reduced products, such as CH_4_, C_2_H_4_, acetate, ethanol and n-propanol, gradually increased and evolved when the applied potential increased, indicating that a higher potential is beneficial to multiple proton-coupled electron-transfer processes at the expense of CO. A similar consumption trend of *FE*_CO_ is also observed in both Me-GDY/Cu NW and F-GDY/Cu NW, revealing its key intermediate role for more reduced product formation. It is worthwhile to point out that the *FE* distribution for those more reduced products varied significantly among the as-prepared catalysts. In Me-GDY/Cu NW, CH_4_ selectivity was drastically boosted as the potential shifted negatively, reaching a $F{{E}_{C{{H}_4}}}$ value of 39.6% at −1.2 V, which is 4.39 times higher than that of Cu NW. In stark contrast, F-GDY/Cu NW substantially impeded CH_4_ formation while favoring C_2_H_4_ production. As shown in Fig. 3e, the C_2_H_4_ derived by F-GDY/Cu NW increased rapidly against the applied potential, rendering an impressive $F{{E}_{{{{\mathrm{C}}}_2}{{{\mathrm{H}}}_{4{\mathrm{\ }}}}}}$ value of 40.9%, which was 1.85 and 1.98 times as high as that of Cu NW and Me-GDY/Cu NW, respectively. Hence, by incorporating surface electron-withdrawing or -donating R-GDY, a significant alteration was observed in the CO intermediate-based C–C coupling and protonation process. Representative NMR results of the prepared Cu NW, F-GDY/Cu NW and Me-GDY/Cu NW electrodes operated at −1.2 V are shown in Figs S8–S10. Furthermore, Fig. 3f summarizes and compares the *FE* of C_2+_ products for the catalysts at various applied potentials. Obviously, the F-GDY/Cu NW contributed significantly higher C_2+_ production than the other two samples. In terms of the C > 2e^−^ products (e.g. CH_4_, C_2_H_4_, ethanol, acetate and n-propanol, collectively denoted as C > 2e^−^), both F-GDY/Cu NW and Me-GDY/Cu NW exhibited higher $F{{E}_{{\mathrm{C}} > 2{{e}^ - }}}$ compared with bare Cu NW (Fig. 3g). Taken together, it can be concluded that the surface functionality of R-GDY was conducive to the multielectron process involving CO intermediates on Cu NW, leading to the formation of more reduced products. To specify, the electron-withdrawing group in F-GDY/Cu NW is preferred for the C–C coupling process, whereas the electron-donating group in Me-GDY/Cu NW aids in the CO protonation process (Fig. 3h).

To rule out surface hydrophobicity as a potential contributor to product variation, contact angle measurements were performed. Figure S11 illustrates that all catalysts exhibited highly hydrophobic surfaces, with contact angle values exceeding 100°, revealing that hydrophobicity does not contribute to distinct product formation over the catalyst surface. Furthermore, long-term electrolysis at a stationary potential of −1.1 V was conducted to assess the durability of R-GDY/Cu NW. Figures S12 and S13 show no significant decrease in overall current density over the electrolysis course. Notably, the slight decrease of $F{{E}_{{\mathrm{C}}{{{\mathrm{H}}}_4}}}$ and${\mathrm{\ }}F{{E}_{{{{\mathrm{C}}}_2}{{{\mathrm{H}}}_4}}}$ over Me-GDY/Cu NW and F-GDY/Cu NW, respectively, is attributed to the flooding during the long-term electrolysis. Comprehensive characterization using HAADF-STEM and EDS confirmed the well-preserved structure and morphology of the R-GDY-coated Cu NW in post-catalysed F-GDY/Cu NW and Me-GDY/Cu NW (Figs S14 and S15).

**Figure 4.**
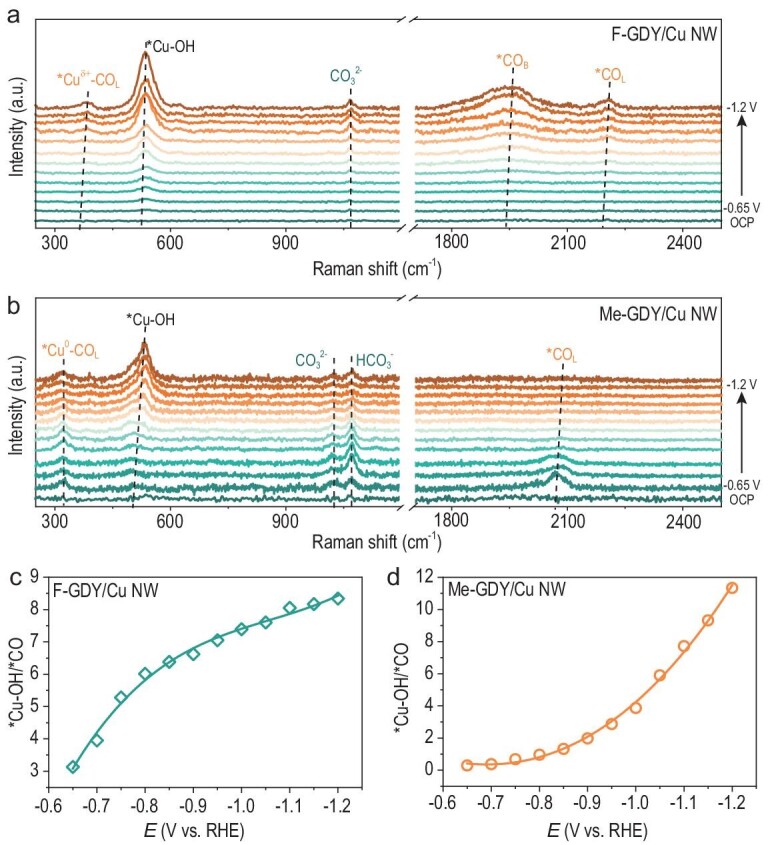
Operando electrochemical SERS measurements. SERS spectra of (a) F-GDY/Cu NW and (b) Me-GDY/Cu NW at a potential range of −0.65 to −1.2 V. Plot of *Cu–OH/*CO ratio versus applied potentials for (c) F-GDY/Cu NW and (d) Me-GDY/Cu NW.

To attain a molecular-level understanding of how the surface functionalities of R-GDY impact CO_2_RR formation, operando surface-enhanced Raman spectroscopy (SERS) was conducted in a spectroelectrochemical flow cell (Fig. S16). Figure 4a and b shows the SERS of F-GDY/Cu NW and Me-GDY/Cu NW as a function of applied potentials during CO_2_RR. Notably, the adsorption of *CO intermediate differs between F-GDY/Cu NW and Me-GDY/Cu NW. A band associated with linearly adsorbed CO (CO_L_) was observed between 2190 and 2210 cm^−1^ on F-GDY/Cu NW, which shifted towards higher wave numbers compared with Me-GDY/Cu NW (2060–2075 cm^−1^) [35]. The positive shift in the CO_L_ band was also mirrored in the Cu-CO_L_ band, with F-GDY/Cu NW positioned at 370–383 cm^−1^, which was higher than Me-GDY/Cu NW, at 315–325 cm^−1^ [36]. The variation in the CO_L_ position was attributed to the fine-tuned interfacial oxidation state of Cu NW by electron-withdrawing (F-GDY) and electron-donating (Me-GDY) group-functionalized R-GDY, respectively. Analysis from the aforementioned XPS and XANES deduced that F-GDY/Cu NW possesses a higher oxidation state of Cu than Me-GDY/Cu NW, leading to the stronger *CO_L_ adsorption and an upward shift in the Raman wave number. Besides, the higher Cu oxidation state manifested the exclusive observed stable bridge-adsorbed CO (CO_B_) at 1945 cm^−1^ on F-GDY/Cu NW, which was a pivotal factor contributing to C_2+_ production [37]. Given the presence of stronger adsorbed *CO_L_ and *CO_B_, their corresponding peak intensities increased as more negative potentials were applied, signifying the enhanced *CO coverage on the F-GDY/Cu NW surface. The enriched *CO coverage was believed to facilitate the C–C coupling process for C_2+_ production, aligning with the observed CO_2_RR behaviors for F-GDY/Cu NW. In contrast, the *CO_L_ peak gradually diminished with a potential shift negatively, suggesting low *CO coverage on the Me-GDY/Cu NW surface. This can be attributed to the weak binding of *CO_L_ to the low-valence state of the Cu surface. Collectively, the exquisite perturbated interfacial Cu oxidation state on R-GDY/Cu NW led to distinct *CO adsorption and coverage, thus, in turn, steering the CO_2_RR pathways.

Of note, a prominent peak centered at 525 cm^−1^ steadily increased within the potential range of −0.65 to −1.2 V on R-GDY/Cu NW, which can be assigned to the *Cu–OH band [38]. Previous studies suggest that the presence of *Cu–OH is conducive to promoting the protonation process during the CO_2_RR, thus facilitating the production of more reduced products [39–42]. The aforementioned CO_2_RR performance clarifies that the primary protonation products over F-GDY/Cu NW and Me-GDY/Cu NW are C_2_H_4_ and CH_4_, respectively. We thus envisioned that the protonation of distinctly adsorbed *CO by *Cu–OH gave rise to diverse product formations on the as-prepared R-GDY/Cu NW. As such, the *Cu–OH/*CO area ratio was determined for both F-GDY/Cu NW and Me-GDY/Cu NW, as shown in Fig. 4c and d, respectively. Encouragingly, the corresponding profiles follow an analogous increasing trend, as observed for $F{{E}_{{{{\mathrm{C}}}_2}{{{\mathrm{H}}}_4}}}$ and $F{{E}_{{\mathrm{C}}{{{\mathrm{H}}}_4}}}$ in F-GDY/Cu NW and Me-GDY/Cu NW, respectively. Hence, the *Cu–OH/*CO area ratio was inferred as a descriptor to elucidate the distinct protonation products over varying oxidation states of the Cu surface. Furthermore, the local pH could be assessed by examining the relative ratio of the pH-dependent HCO_3_^−^/CO_3_^2−^ equilibrium (HCO_3_^−^ at 1015 cm^−1^, CO_3_^2−^ at 1065 cm^−1^) [43,44]. Analysis of the recorded Raman spectra revealed a prominent HCO_3_^−^ band for Me-GDY/Cu NW across the applied potential range, whereas it was barely discernible for F-GDY/Cu NW. This underscored the lower local pH of Me-GDY/Cu NW compared with that of F-GDY/Cu NW during the CO_2_RR. Generally, a lower local pH favors CH_4_ production, while a higher local pH promotes CO and/or C_2_H_4_ production [45], which is in line with our findings.

**Figure 5.**
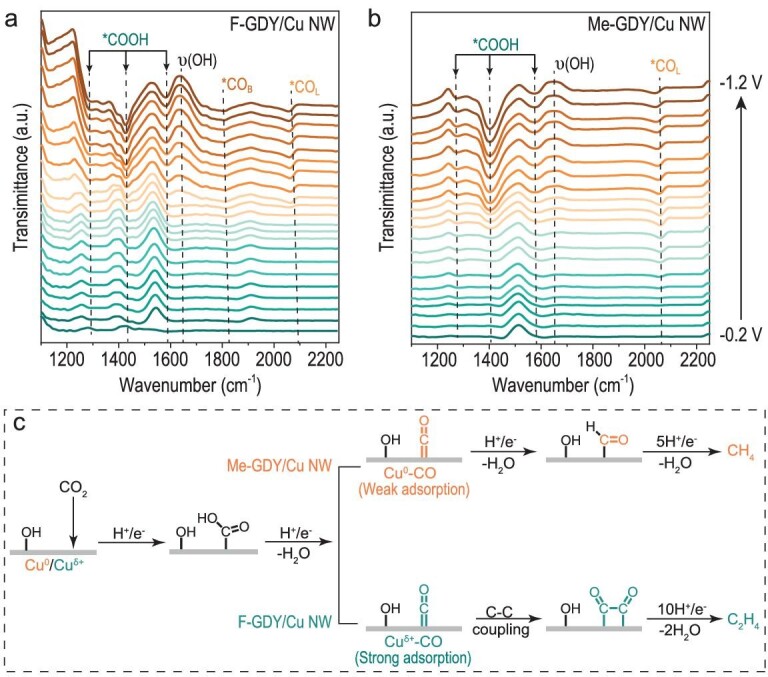
*In situ* ATR-FTIR measurements and reaction pathways. *In situ* ATR-FTIR spectra of (a) F-GDY/Cu NW and (b) Me-GDY/Cu NW at a potential range of −0.2 to −1.2 V. (c) Proposed reaction pathways for C_2_H_4_ and CH_4_ formation over F-GDY/Cu NW and Me-GDY/Cu NW, respectively.

We further investigated CO_2_RR intermediates through *in situ* attenuated total reflection FTIR (ATR-FTIR). Figure 5a and b depicts the ATR-FTIR spectra of F-GDY/Cu NW and Me-GDY/Cu NW within a potential range from −0.2 to −1.2 V. Four discernible peaks at 1250, 1334, 1400 and 1560 cm^−1^ correspond to the OH deformation, C−O stretch, symmetric stretch and asymmetric stretch of *COOH, respectively, which is widely recognized as a pivotal intermediate in the formation of CO [46,47]. Notably, a positive shift in the *CO_L_ peak was observed, transitioning from ∼2080 cm^−1^ in F-GDY/Cu NW to ∼2055 cm^−1^ in Me-GDY/Cu NW [48]. Moreover, a *CO_B_ band at ∼1830 cm^−1^ was exclusively observed on F-GDY/Cu NW, which echoes the above operando Raman results. Taken together, the adsorbed CO_2_ underwent a sequential hydrogenation process, forming *COOH followed by the *CO intermediates. Specifically, the higher oxidation state of the Cu interface tailored by the electron-withdrawing F-GDY showcased a stronger *CO adsorption compared with the electron-donating Me-GDY regulated interface. The enhanced *CO adsorption enriched the coverage on F-GDY/Cu NW, thereby facilitating the C–C coupling process. Meanwhile, the presence of *Cu–OH species facilitated the protonation process, leading to the formation of C_2_H_4_ and CH_4_ products on F-GDY/Cu NW and Me-GDY/Cu NW, respectively (Fig. 5c).

**Figure 6.**
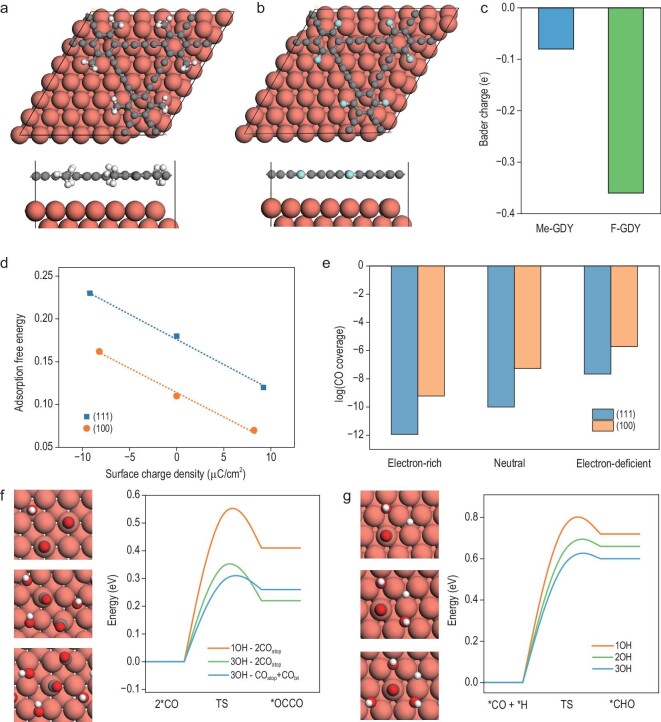
DFT calculation results. The model structures of (a) Me-GDY/Cu (111) and (b) F-GDY/Cu (111). (c) Bader charge analysis of Cu (111) surface when covered with Me-GDY and F-GDY. (d) Adsorption free energy of CO on Cu (111) and Cu (100) under different charge conditions; the pressure of CO is 0.05 atm in this work, which corresponds to the CO partial pressure observed in the CO_2_RR on polycrystalline Cu [49]. (e) Predicted CO coverage under different charge conditions using the Langmuir adsorption model. (f) C–C coupling barriers under different OH coverage and CO adsorption modes. (g) *CO hydrogenation to *CHO barriers under different OH coverage.

Density functional theory (DFT) calculations were conducted to gain an insight into the molecular-level mechanisms involved. We specifically focused on elucidating the electron-push/pull effects of fluoro- and methyl-functionalized graphdiyne (F-GDY and Me-GDY, respectively) on copper surfaces. For this purpose, models of F/Me-GDY-covered Cu (111) surfaces were constructed (Fig. 6a and b). Bader charge analysis revealed that both F-GDY and Me-GDY acquired a certain number of electrons from the Cu surface. However, the extent of electron depletion on the Cu surface differed notably between F-GDY/Cu (111) and Me-GDY/Cu (111). While the Cu surface covered with Me-GDY showed a depletion of only 0.09 e^−^, the electron transfer was significantly more pronounced with F-GDY, leading to an electron depletion of ≤0.36 e^−^ on Cu (111) (Fig. 6c). This electron transfer indicated that the Cu surface assumed an electron-deficient state, particularly when covered with F-GDY.

Subsequently, we investigated how these electron-rich or electron-deficient states affected the CO adsorption strength. Due to the large size of the F-GDY/Cu (111) and Me-GDY/Cu (111) systems, further calculations were performed on smaller (3 × 3) Cu (111) and Cu (100) surfaces to qualitatively deduce trends. It was observed that the CO adsorption energy was influenced by the surface charge density. Specifically, enriching the Cu surface with 0.5 electrons (corresponding to a negative surface charge density) weakened the CO adsorption strength. Conversely, removing 0.5 electrons strengthened the adsorption (Fig. 6d). These trends were consistent across both Cu (111) and Cu (100) surfaces, suggesting that the phenomenon was not structure-dependent. This aligns with experimental findings from Operando SERS measurements. Furthermore, employing the Langmuir adsorption model for predicting CO coverage at varying electron concentrations revealed that an electron-deficient surface enhanced CO adsorption and increased CO coverage (Fig. 6e). It is important to note that these coverage predictions are qualitative rather than quantitative.

*In situ* ATR-FTIR spectroscopy results underscored the significance of surface OH groups in catalytic activity. DFT calculations indicated that higher OH coverage fostered C–C coupling, as observed when comparing conditions with one and three OH groups. Additionally, it was found that coupling between one atop-bound CO molecule (CO_atop_) and one bridge-bound CO molecule (CO_bri_) resulted in a lower energy barrier, even though the reaction energy did not decrease significantly (Fig. 6f). This finding is in agreement with experimental observations that the presence of bridge-site CO facilitates the formation of C_2_ products. Moreover, the level of OH coverage also impacted the CO hydrogenation elementary step, with higher OH coverage promoting CO hydrogenation, as illustrated in Fig. 6g.

## CONCLUSION

In this study, we have achieved distinct electronic perturbation of the Cu surface through the modification of Cu NW with electron-withdrawing/-donating groups-functionalized R-GDY (R = –F and –Me), presenting a compelling approach to steer the CO_2_RR pathway. Comprehensive analyses revealed that the electron density of the Cu NW surface was perturbated by the surrounding R-GDY, resulting in a higher Cu oxidation state for F-GDY/Cu NW compared with Me-GDY/Cu NW. *In situ* spectroscopical studies unraveled that the binding strength of *CO and *CO coverage scaled directly with the Cu surface oxidation state of R-GDY/Cu NW, leading to a more favorable C–C coupling process on F-GDY/Cu NW than on Me-GDY/Cu NW. As a result, F-GDY/Cu NW showcased a preferential production of C_2_H_4_ with a remarkable selectivity of 73.15% ± 2.5% for C_2+_ products, while Me-GDY/Cu NW predominantly favored CH_4_ formation. Furthermore, utilizing the *Cu–OH/*CO ratio as a descriptor, the mechanistic study demonstrated that the protonation of distinctly adsorbed *CO facilitated by *Cu–OH contributed to the resultant favorable C_2_H_4_ and CH_4_ production on F-GDY/Cu NW and Me-GDY/Cu NW, respectively. This study provides valuable guides into the exquisite perturbation of the electronic structure of Cu surfaces, holding great potential for advancing catalyst design in the CO_2_RR.

## Supplementary Material

nwae253_Supplemental_File
