# Supplementary material for: Electronic perturbation of Cu nanowire surfaces with functionalized graphdiyne for enhanced CO2 reduction reaction
Source: Natl Sci Rev. 2024 Jul 25;11(12):nwae253. doi: 10.1093/nsr/nwae253 (PMC11562832; doi:10.1093/nsr/nwae253)
Supplement: nwae253_Supplemental_File [file nwae253_supplemental_file.docx]

**Supplementary data**

**Electronic perturbation of Cu nanowire surfaces with functionalized graphdiyne for enhanced CO_2_ reduction reaction**

Haiyuan Zou,^1,3,†^ Dongfang Cheng,^3,†^ Chao Tang,^1,†^ Wen Luo,^4,†^ Huatian Xiong,^2^ Hongliang Dong,^5^ Fan Li,^1^ Tao Song,^1^ Siyan Shu,^1^ Hao Dai,^1^ Zhouguang Lu,^4^ Lele Duan^1,2,6,7^*

^1^Department of Chemistry, Southern University of Science and Technology, Shenzhen, 518055, P. R. China

^2^Center of Artificial Photosynthesis for Solar Fuels and Department of Chemistry, School of Science, Westlake University, 310024 Hangzhou, China

^3^Departmentof Chemical and Biomolecular Engineering, University of California Los Angeles, CA 90095, USA

^4^Department of Materials Science and Engineering, Southern University of Science and Technology, Shenzhen 518055, China

^5^Center for High-Pressure Science and Technology Advanced Research, Pudong, Shanghai 201203, China.

^6^Division of Solar Energy Conversion and Catalysis at Westlake University, Zhejiang Baima Lake Laboratory Co., Ltd, Hangzhou 310000, Zhejiang, China

^7^Institute of Natural Sciences, Westlake Institute for Advanced Study, Hangzhou 310024, China;

**∗Corresponding authors.** E-mails: duanlele@westlake.edu.cn

†Equally contributed to this work.

**Materials:** Pyridine, acetone, and tetrabutylammonium fluoride (TBAF, 1.0 M in THF) were sourced from Energy Chemical. Hydrazine hydrate (90% in water), sodium hydroxide, and ethylenediamine were obtained from Sigma Aldrich. The water used was purified using a Millipore system with a typical resistivity of 18.2 MΩ cm. Gas diffusion layers (GDL) were procured from Fuel Cell Store, specifically the Sigracet 29 BC. The counter electrode, an IrO_2_-coated titanium sheet, was purchased from Baoji Zhiming Special Metal Co., LTD. All other chemicals used are commercially available without further purification. 2,4,6-Tris(trimethylsilylethynyl)-1,3,5-trifluorobenzene, 2,4,6-triiodomesitylene, 1,3,5-tris(trimethylsilylethynyl)mesitylene were prepared according to the literature methods.

**Preparation of Cu nanowires (Cu NW).** Cu NW was synthesized using a previously reported method.[1] Initially, 250 g of NaOH was dissolved in 380 mL of deionized water under an ice bath. Subsequently, a solution of Cu(NO_3_)_2_ (1.23 g, 6.56 mmol) pre-dissolved in 5 mL of deionized water was added dropwise to the above solution after returning to room temperature. Once the system achieved uniform dispersion, ethylenediamine (3 mL) and hydrazine hydrate (300 μL) were introduced. The mixture was stirred at room temperature for 30 minutes and then transferred to an oven at 80 ℃ for 1 hour. The resulting precipitate was collected through centrifugation, washed with water and ethanol, and subsequently vacuum-dried overnight, yielding Cu nanowires in the form of reddish-brown powders.[2-4]

**Preparation of 2,4,6-Tris(trimethylsilylethynyl)-1,3,5-trifluorobenzene.** According to the literature methods,[2] to a 100 mL three-necked flask filled with nitrogen was added 2,4,6-Triiodo-1,3,5-trifluorobenzene (2 g, 3.9 mmol), PdCl_2_(PPh_3_)_2_ (0.275 g, 0.4 mmol), CuI (75 mg, 0.4 mmol) and Et_3_N (20 mL). A solution of trimethylsilyl acetylene (1.35 g, 13.6 mmol, 3.5 eq) in Et_3_N (10 mL) was added dropwise. At the end of the addition, the mixture was warmed up to 70 ℃. THF (10 mL) was added after 1 h, and the mixture was left stirring for 16 h. The mixture was filtered over Celite and purified by column chromatography on silica gel using hexane as an eluant. The product was recrystallized in hexane to afford 0.7 g (1.7 mmol, 42%) white solid. ^1^H NMR (400 MHz, CDCl_3_): δ 0.26 (27 H, s, CH_3_).^13^C NMR (100 MHz, CDCl_3_): δ 163.57 (t, ^1^*J*_CF_ = 261 and ^3^*J*_CF_ = 7 Hz), 107.15 (m), 99.70 (m), 88.96 (s), 0 (s). ^19^F NMR (376 MHz, CDCl_3_): δ -99.37.

**Preparation of** **2,4,6-triiodomesitylene.** According to the literature methods,[3] to a 100 mL three-necked flask filled with nitrogen was added iodine (3.81 g, 15 mmol) and carbon tetrachloride (15 ml), followed by the addition of [Bis(trifluoroacetoxy)iodo]benzene (7.1 g, 16.5 mmol) and mesitylene (1.2 g, 10 mmol). The mixture was left stirring for 5 h at room temperature. The precipitate was filtered, washed with a small volume of carbon tetrachloride and dried under vacuum to give 4.2 g (8.44 mmol, 84%) white solid. ^1^H NMR (400 MHz, CDCl_3_): δ 3.01 (9H, s, Ar-CH_3_).

**Preparation of 1,3,5-tris(trimethylsilylethynyl)mesitylene.** According to the literature methods,[4] 2,4,6-triiodomesitylene (500 mg, 1 mmol), PdCl_2_(PPh_3_)_2_ (0.275 g, 0.4 mmol) and CuI (75 mg, 0.4 mmol) were placed in a three-necked flask that filled with nitrogen, followed by addition of Et_3_N (10 mL). A solution of trimethylsilyl acetylene (588 g, 6 mmol, 6 eq) in Et_3_N (5 mL) was added dropwise. At the end of the addition, the mixture was stirred at room temperature. After 1h, THF (3 mL) was added, and the mixture was left stirring for 72 h. The mixture was filtered over Celite and purified by column chromatography on silica gel using hexane as an eluant. The product was recrystallized in hexane to afford 328 mg (0.8 mmol, 80%) white solid. ^1^H NMR (400 MHz, CDCl_3_): δ 2.55 (9H, s, Ar-CH_3_), 0.26 (27H, s, CH_3_).

**Preparation of R-GDY/Cu NW (R = –F/–Me).** To synthesize R-GDY/Cu NW, 30 mg of Cu NW and R-GDY monomers (0.122 mmol) were blended in a 100 mL round bottom flask under a dry, oxygen-free argon atmosphere. Pyridine (6 mL) and acetone (50 mL) were subsequently added. After 30 minutes of sonication, 1 mL of TBAF (1.0 M in TBAF, 0.4 mmol) was introduced. The system was then heated to 40 ℃ for 24 hours. The resulting precipitate was isolated through centrifugation, followed by washing with ethanol and vacuum drying.

**Preparation of gas diffusion electrodes (GDEs).** GDEs were prepared by employing a drop-coating technique to load catalysts onto a gas diffusion layer sheet. The ink formulation consisted of 2 mg of catalyst (Cu NW, F-GDY/Cu NW, Me-GDY/Cu NW), 40 μL of 5 wt% Nafion solution (Sigma–Aldrich), 0.8 mL of pure water, and 0.2 mL of alcohol. This mixture was sonicated for 1 h to form a homogeneous ink before drop-coating. The resulting well-dispersed ink was applied onto a gas diffusion layer piece (1 × 3 cm²) with a catalyst-coated area of (0.5 × 2 cm²). The gas diffusion layer was then dried in a vacuum chamber prior to use. The loading amount was determined by weighing the gas diffusion layer before and after drop coating, and was found to be approximately 0.5 mg cm^–2^.

**Flow-cell CO_2_ electrolysi**s. CO_2_ reduction was performed in a three-chamber flow cell with channels measuring 2 cm × 0.5 cm × 0.15 cm. The gas flow was regulated by an ALICAT mass flow controller set at 20 sccm. A 99.99% aqueous KOH solution (Sigma–Aldrich) served as both catholyte and anolyte, with peristaltic pumps maintaining a flow rate of 4.6 mL min^−1^. A FAA–3 (Fumatech) hydroxide exchange membrane separated the cathode and anode chambers. The counter electrode consisted of an IrO_2_-coated titanium sheet. Electrolysis experiments were conducted using chronopotentiometry with CHI 760 potentiostat. Cathode potentials were measured against an external Ag/AgCl reference electrode (saturated 3 M KCl) and then converted to the reversible hydrogen electrode (RHE) scale using the formula E (*vs.* RHE) = E (vs. Ag/AgCl) + 0.196 V + 0.0591 × pH without *iR* correction, where 0.196 corresponds to V^0^_Ag/AgCl vs. NHE_ at 25 °C. For each current density, products were quantified over 300 s, and at least three replicates were performed to obtain an average. Gas products were analyzed using an in-line multiple gas analyzer gas chromatography system equipped with a thermal conductivity detector and a flame ionization detector. Argon (99.999%) served as the carrier gas.

The Faradaic efficiency (FE) of gaseous products was calculated using the equation:

$$FE= \frac{nFxV}{j}$$

where n represents the number of transferred electrons, F is Faraday constant, *x* is the mole fraction of the gas product, *V* is the total molar flow rate of the gas reactant, and *j* is the total applied current during CO_2_RR.

Liquid products were analyzed using a Bruker AVIII 400 MHz NMR spectrometer. In brief, 400 μL of the sampled catholyte was mixed with 50 μL of D_2_O and 50 μL of H_2_O containing 80 ppm (v/v) dimethyl sulfoxide (≥ 99.9% (Alfa Aesar)) as the internal standard. The ^1^H-NMR spectrum was recorded with water suppression method.

The FE of liquid products was calculated using the formula:

$$FE= \frac{nF}{Q}$$

where n represents the mole amount of the product, F is Faraday constant, and Q is the total quantity of electric charge.

**Characterizations.** X-ray photoelectron spectra (XPS) were acquired using an XSAM800 instrument with incident Al *K*α radiation at 1486.6 eV. High-angle annular dark-field scanning transmission electron microscopy and energy-dispersive X-ray spectroscopy mapping were performed on a FEI Titan Themis aberration-corrected transmission electron microscope at 300 kV and FBI Talos transmission electron microscope at 200 kV. FTIR spectra were recorded with a Bruker Vertex 80 infrared spectrometer. X-Ray diffraction patterns were acquired using a Rigaku diffractometer (Japan) with Cu Kα radiation at 1.5418 Å. Surface wettability of the samples deposited on the GDL was assessed using a Kruss DSA 30 contact angle analyzer. X-ray absorption fine structure (XAFS) measurements were carried out on beamline 14W1 at the Shanghai Synchrotron Radiation Facility (SSRF), China. The SSRF storage ring operated at 3.5 GeV with a current of 300 mA. Data analysis was performed using Athena and Artemis software.

***In situ* electrochemical Raman measurements*.*** The in situ Raman measurement was conducted using a three-electrode spectro-electrochemical flow cell reactor. This reactor consists of three channels of anode, cathode, and a gas chamber, which equipped with a quartz window. An FAA-3 hydroxide exchange membrane separates the anode and cathode chambers. CO_2_ gas was introduced into the gas chamber at the back of the gas diffusion layer at a flow rate of 10 sccm, regulated by an ALICAT mass flow controller. The electrolyte, a 0.01 M KOH aqueous solution, was pumped over the GDL at a constant flow rate of 1 mL min^–1^ using a peristaltic pump. The working electrode mirrors the aforementioned GDEs. Counter and reference electrodes were Pt wire and Ag/AgCl (saturated with KCl), respectively. During the in situ Raman tests, Raman spectra were recorded against the applied potential within the range of –0.65 V to –1.2 V *vs.* RHE using a Renishaw Via Raman spectrometer (λ = 532 nm).

***In situ* electrochemical ATR-FTIR measurements*.*** The in situ electrochemical ATR-FTIR measurements were conducted using a Bruker Vertex 80 infrared spectrometer equipped with a liquid nitrogen-cooled RT-DLaTGS detector. Initially, a hemisphere silicon prism was meticulously polished with diamond abrasion paste and then sonicated with deionized water. Subsequently, the prism was immersed in a piranha solution (98% H_2_SO_4_: 30% H_2_O_2_, v/v = 3/1) for 2 hours. Following this, three layers of metal were deposited on the pre-treated silicon prism in the following order: Cr (1.5 nm), Au (45 nm), and Ti (3 nm). This deposition was carried out using an electron beam evaporation system (HHV, TF500). The R-GDY/Cu NW ink slurry was spin-coated onto the metal-deposited silicon prism using a spin coater, which was then assembled into a three-electrode spectroelectrochemical cell as the working electrode. Counter and reference electrodes were Pt wire and Ag/AgCl (saturated with KCl), respectively. A constant flow of CO_2_ was continuously purged into the 0.5 M KHCO_3_ electrolyte throughout the experiment. The in situ FTIR spectra were collected by sweeping the applied potential from –0.2 V to –1.2 V *vs.* RHE at a scan rate of 2 mV s^−1^ with a resolution of 16 s per spectrum at a spectral resolution of 4 cm^−1^.

**Theoretical methods.** All calculations in this work were performed using the Vienna Ab-Initio Simulation Package (VASP)[5], where RPBE functional was used[6]. The core electrons were described with the projector augmented wave (PAW) method[7]. The convergence criteria for electronic and force minimization are set to 10^-6^ eV and 0.02 eV/Å for the optimization. The cutoff energy for the kinetic energy of the plane-waves was 450 eV. ZPE and entropy corrections are determined from frequency calculations using the harmonic oscillator approximation. The Brillouin zone was sampled using the 1 × 1 × 1 and 5 × 5 × 1 Gamma-centered k-point grids, for R-GDY/Cu(111) models and (3*3) Cu(111)/(100) models, respectively. Climbing image nudged elastic band (CI-NEB) method[8] with image-dependent pair potential (IDPP) interpolation[9] was used for searching for the transition states (TS). Each TS geometry has been confirmed to have only one imaginary mode. The Bader charges are calculated using Bader Charge Analysis program[10].


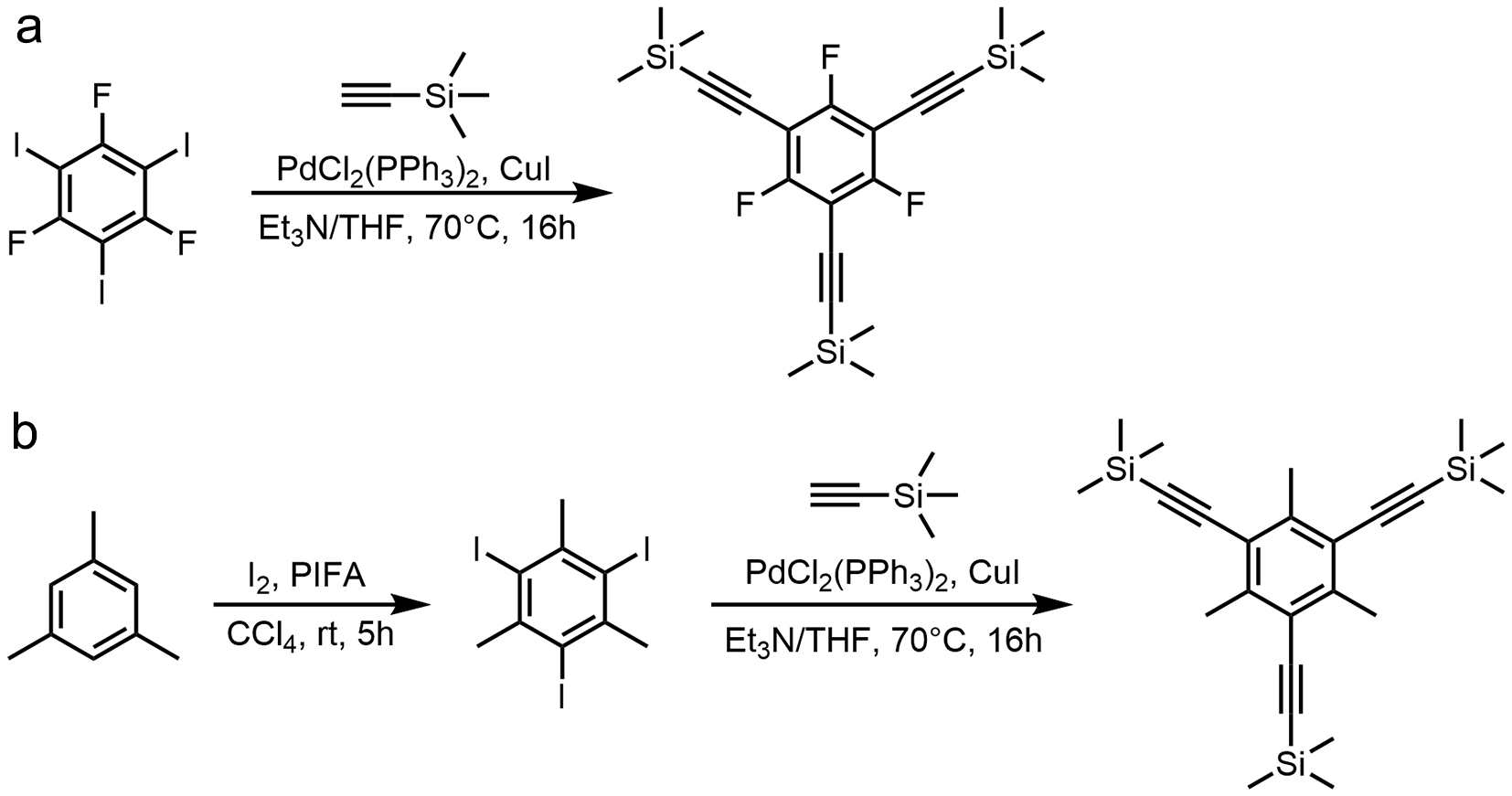


**Scheme S1.** Synthesis procedure of group-modified GDY monomers. (a) 2,4,6-Tris(trimethylsilylethynyl)-1,3,5-trifluorobenzene. (b) 1,3,5-tris(trimethylsilylethynyl)mesitylene.


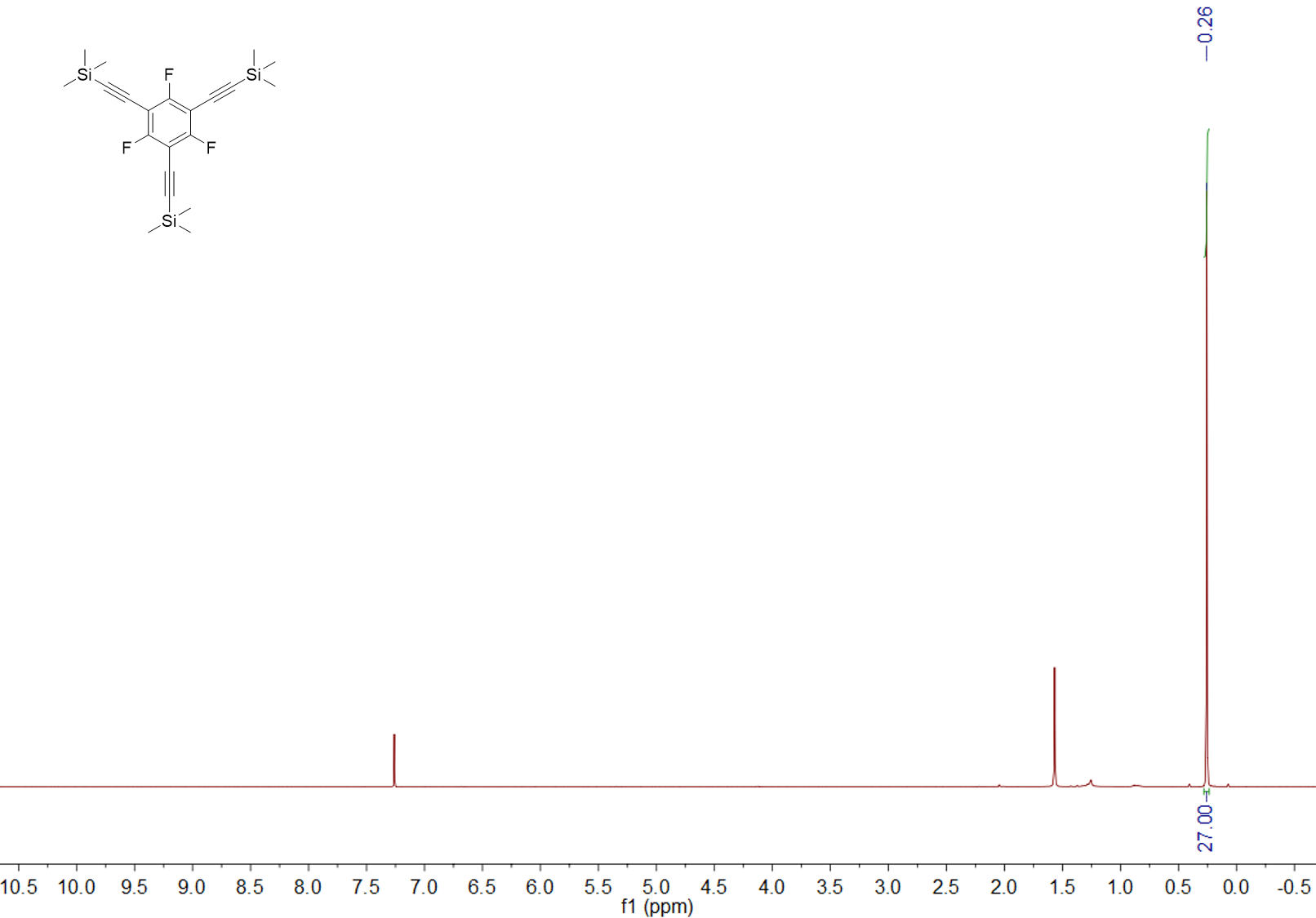


**Figure S1.** ^1^H-NMR (400 MHz, CDCl_3_) spectrum of 2,4,6-Tris(trimethylsilylethynyl)-1,3,5-trifluorobenzene.


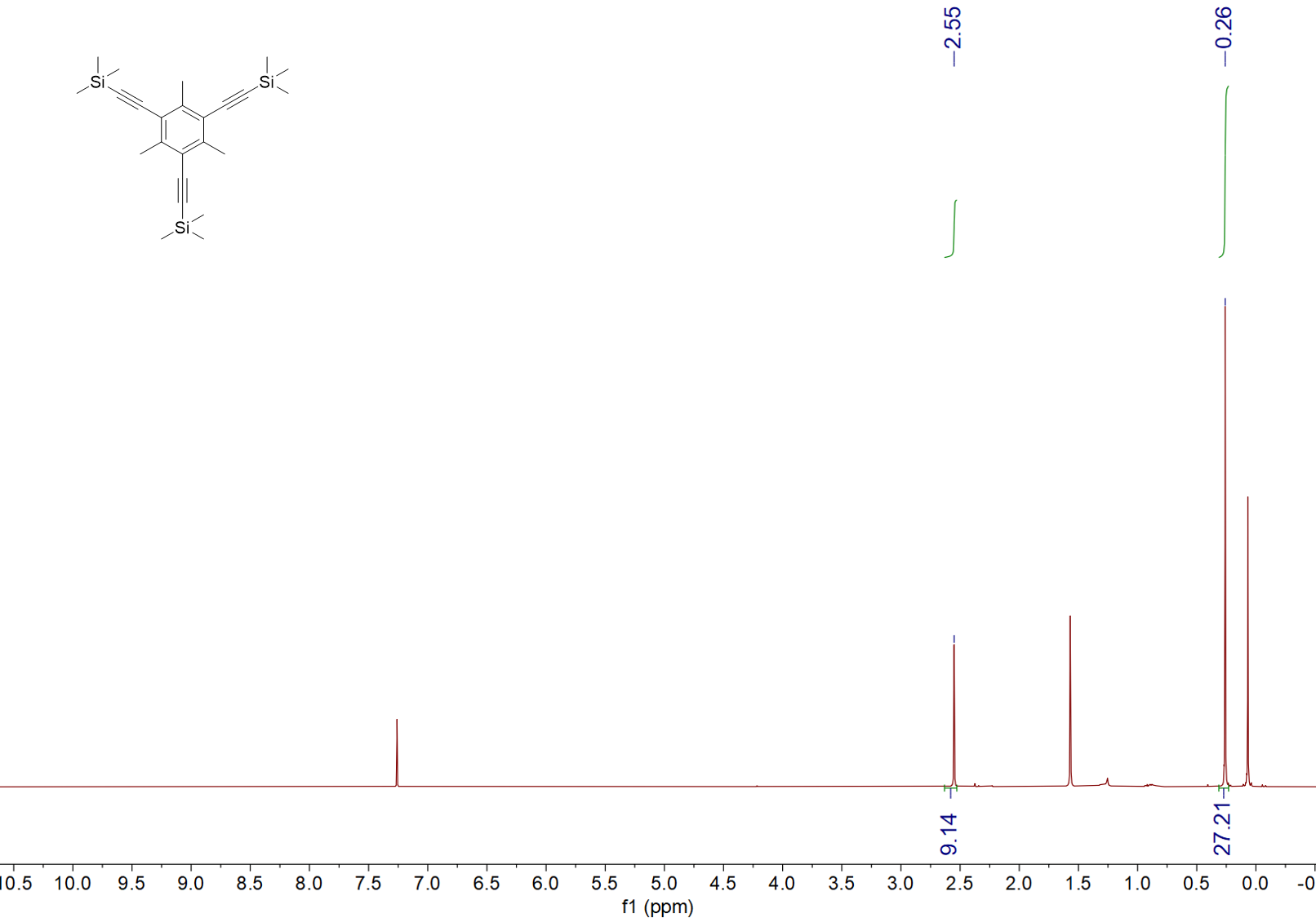


**Figure S2.** ^1^H-NMR (400 MHz, CDCl_3_) spectrum of 1,3,5-tris(trimethylsilylethynyl)mesitylene.


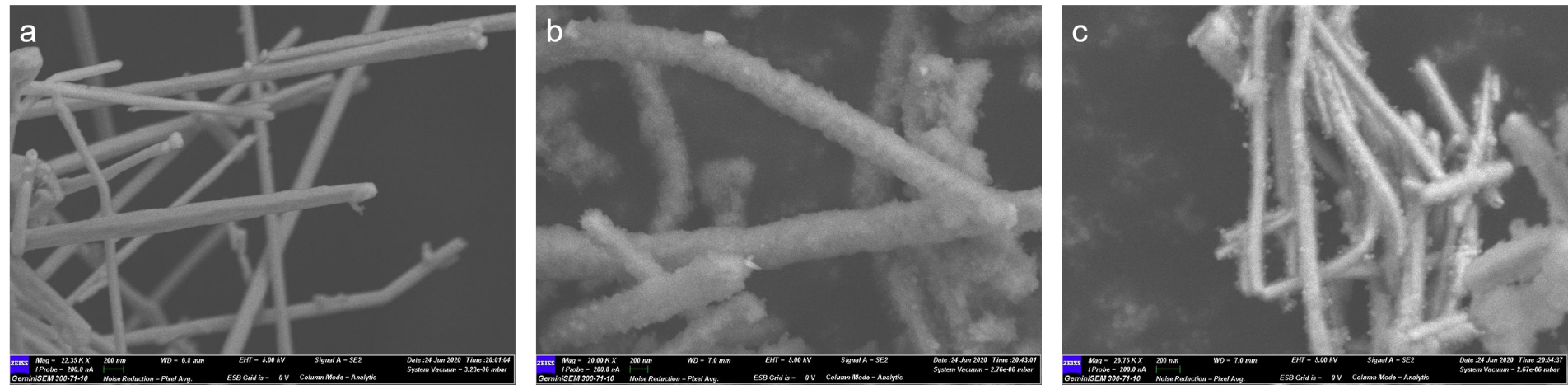


**Figure S3.** SEM images of (a) Cu NW, (b) F-GDY/Cu NW and (c) Me-GDY/Cu NW.

**Table 1.** Surface area of the as-prepared samples.

| Samples | Cu NW | F-GDY/Cu NW | Me-GDY/Cu NW |
| --- | --- | --- | --- |
| *S*_BET_ (m^2^ g^-1^) | 16.94 | 82.34 | 103.47 |


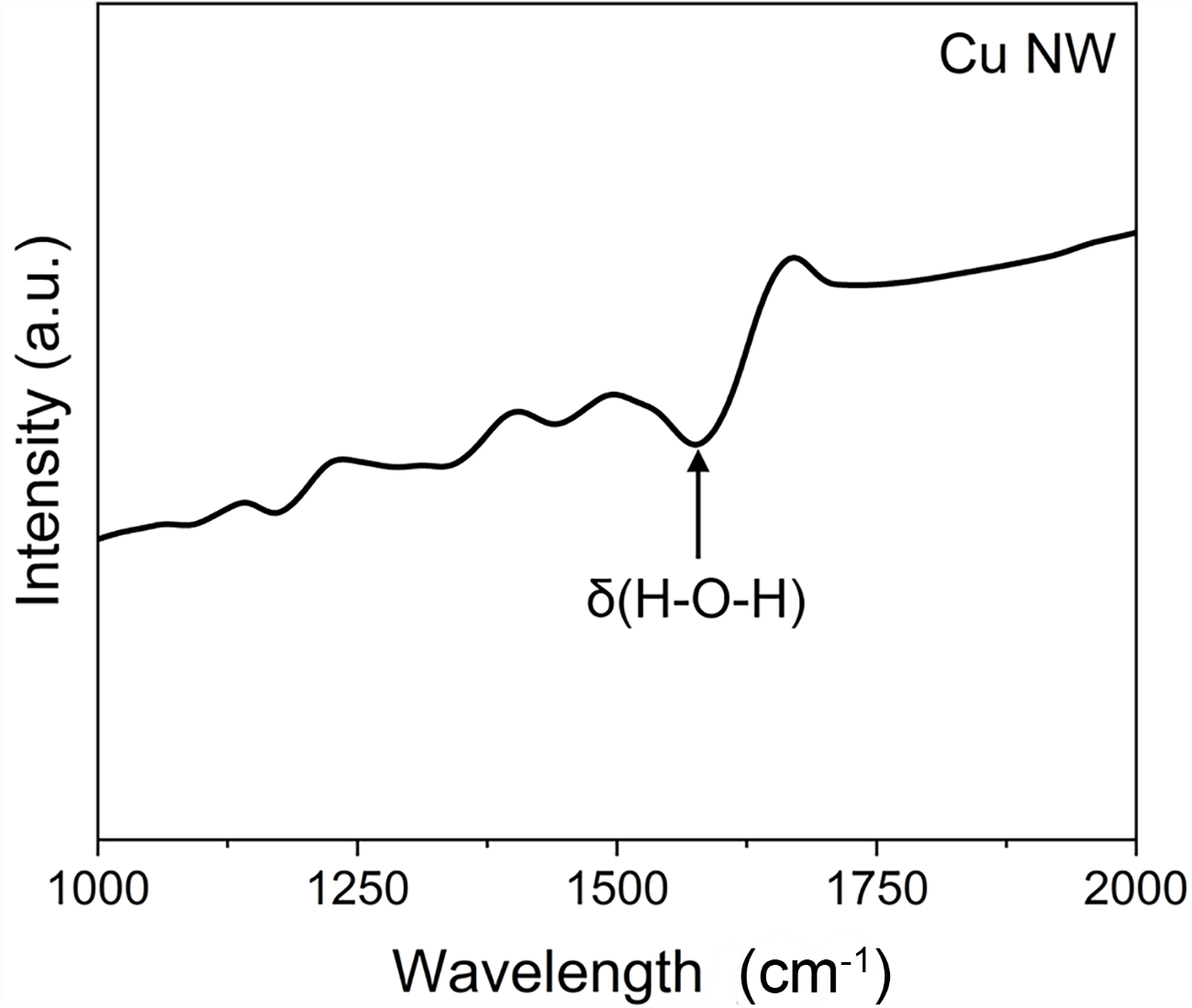


**Figure S4.** FTIR spectra of Cu NW.


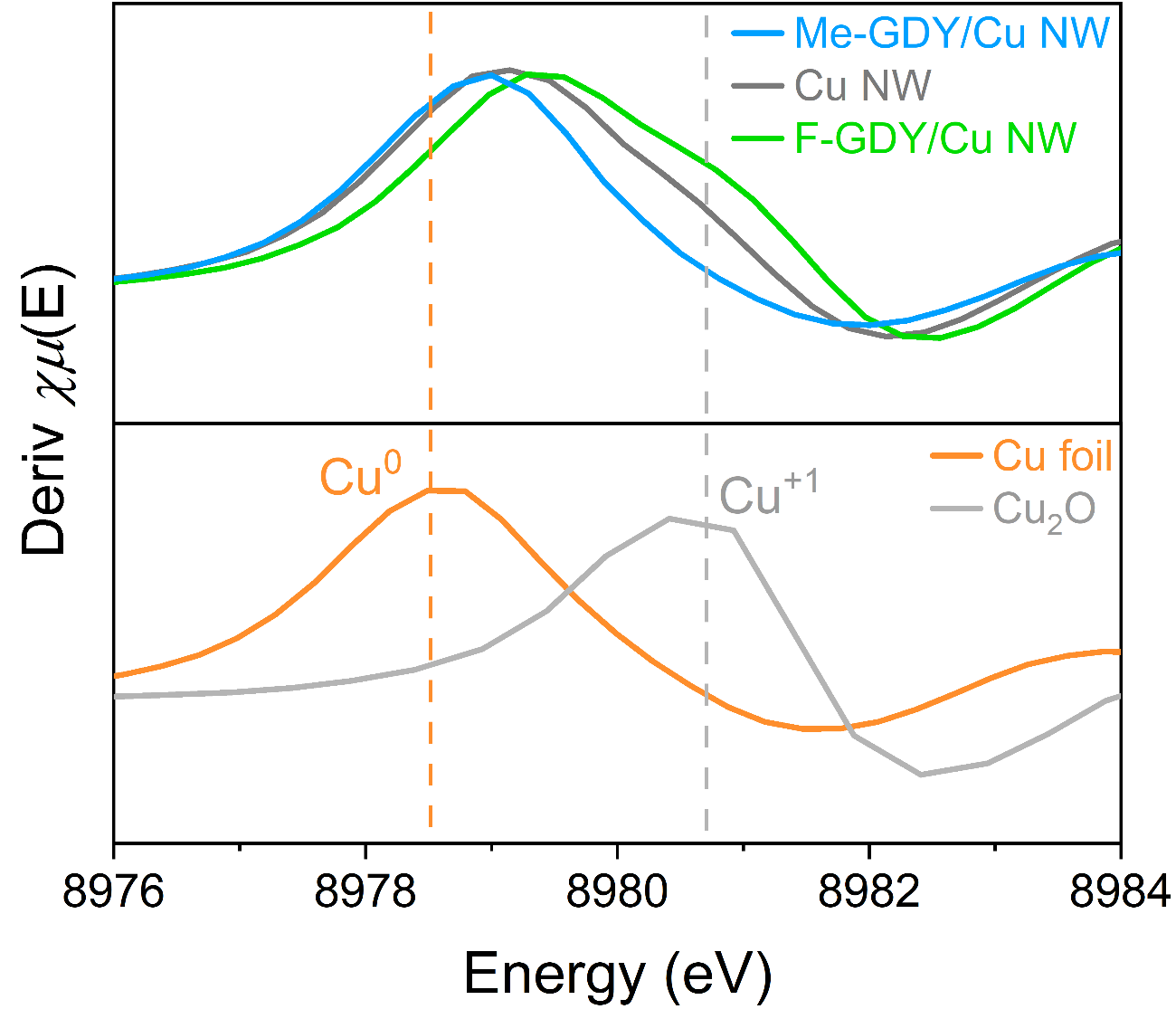


**Figure S5.** First-order derivatives of XANES spectra for Cu NW, F-GDY/Cu NW, Me-GDY/Cu NW, Cu foil and Cu_2_O.


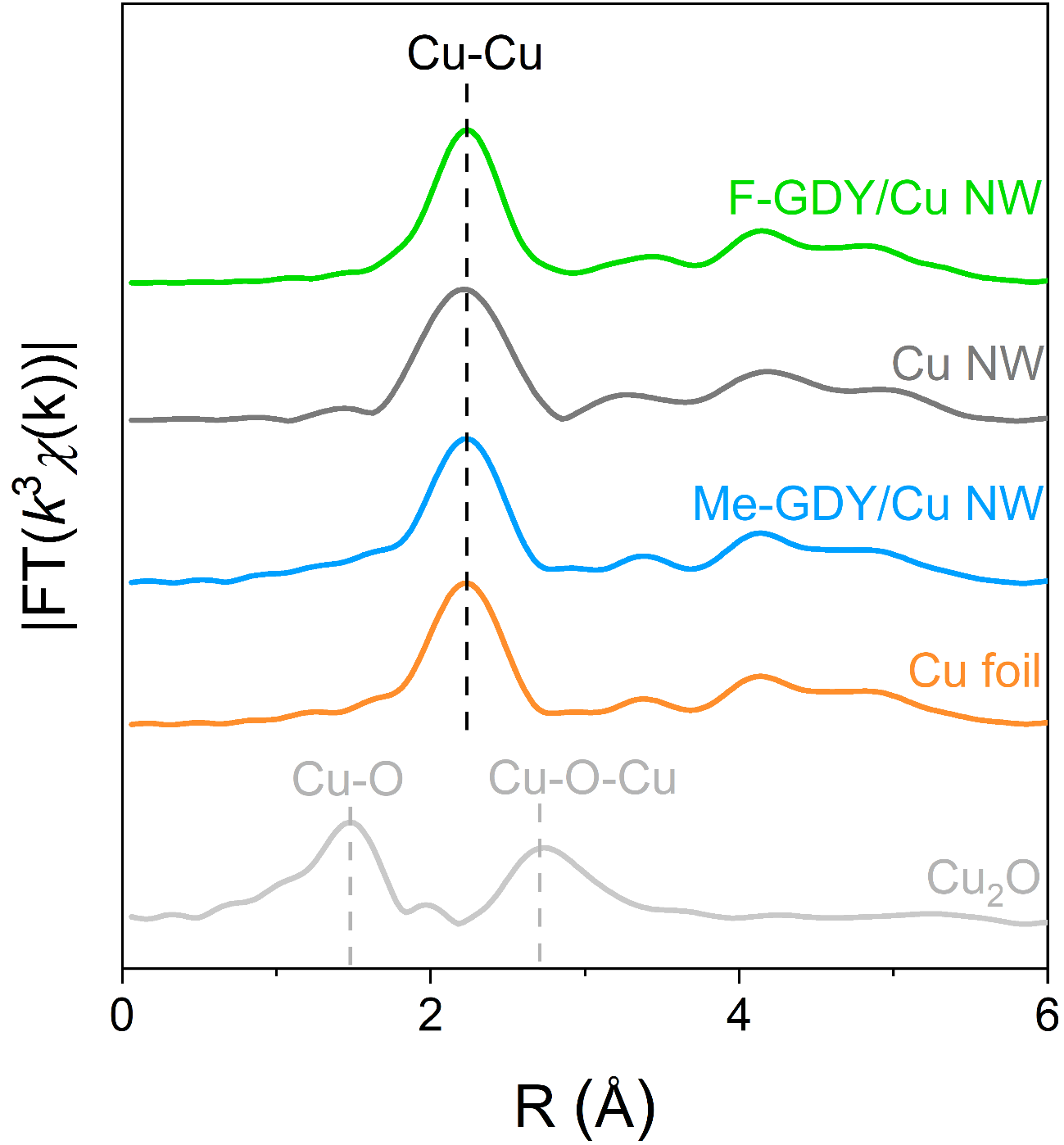


**Figure S6.** Cu *k*^3^-weighted FT-EXAFS spectra in R space of Cu NW, F-GDY/Cu NW, Me-GDY/Cu NW, Cu foil and Cu_2_O.


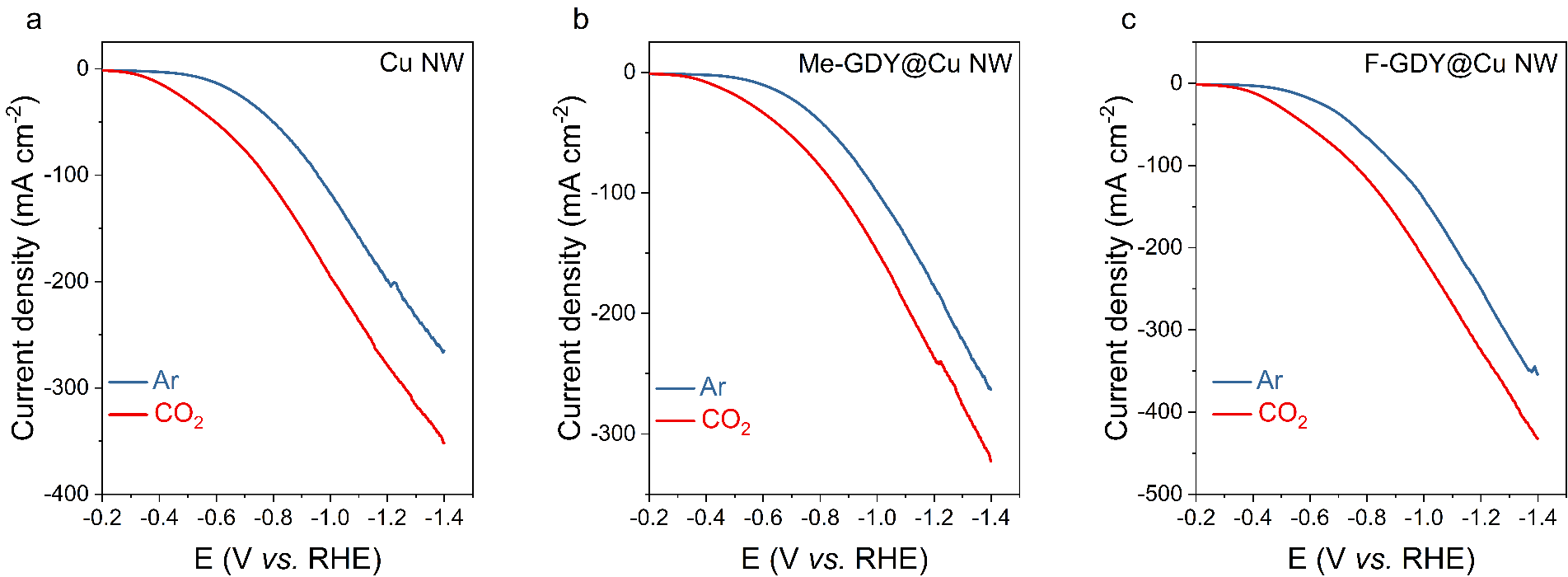


**Figure S7.** LSV curves of as-prepared catalysts in a flow cell reactor. LSV curves of (a) Cu NW, (b) Me-GDY/Cu NW and (c) F-GDY/Cu NW measured in CO_2_-/Ar-flowed 1 M KOH electrolyte.


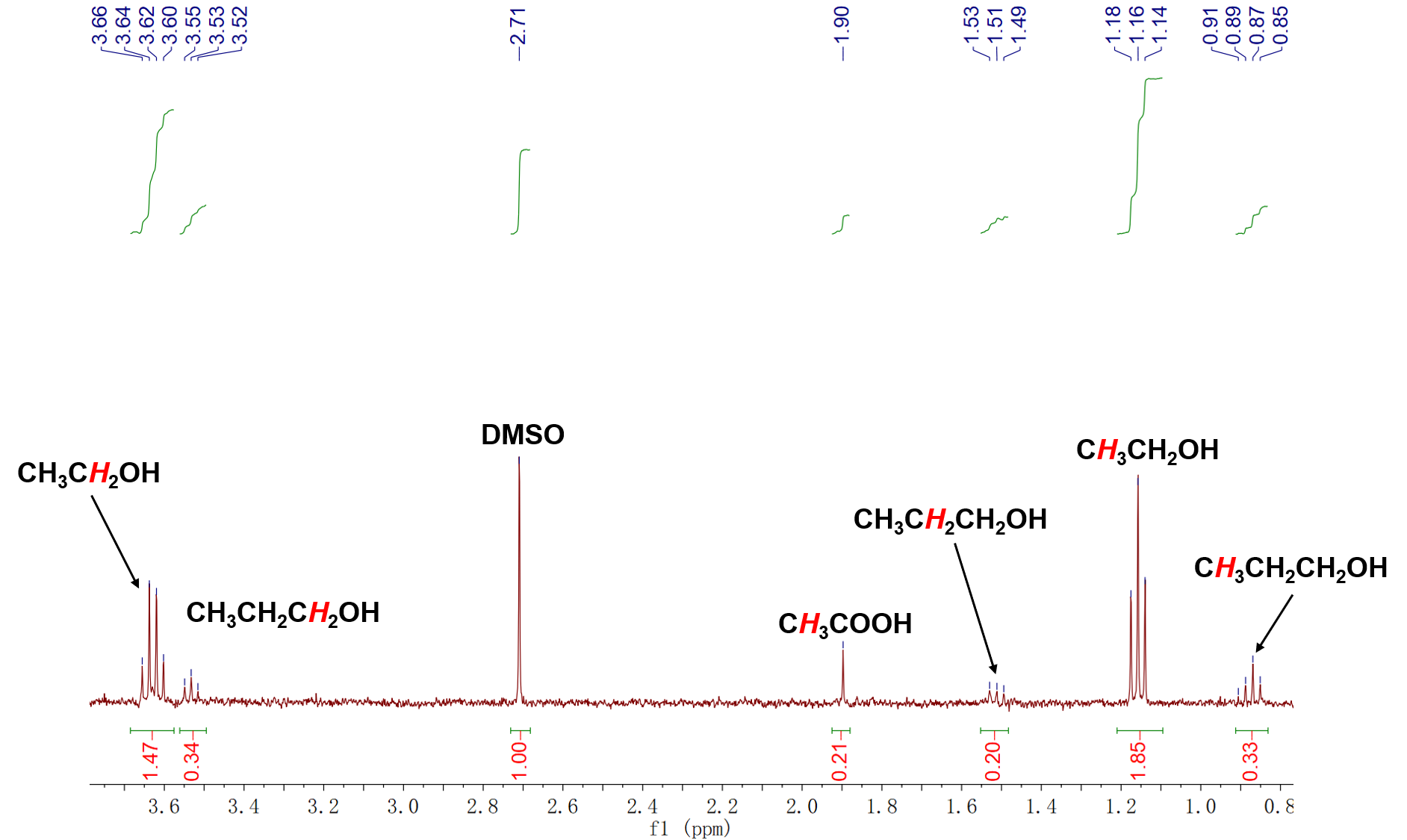


**Figure S8.** ^1^H NMR spectrum (400 M, 10% D_2_O + 90% H_2_O, water suppression, 8 ppm DMSO) of liquid products acquired from Cu NW for CO_2_RR at –1.2 V vs. RHE in 1 M KOH.


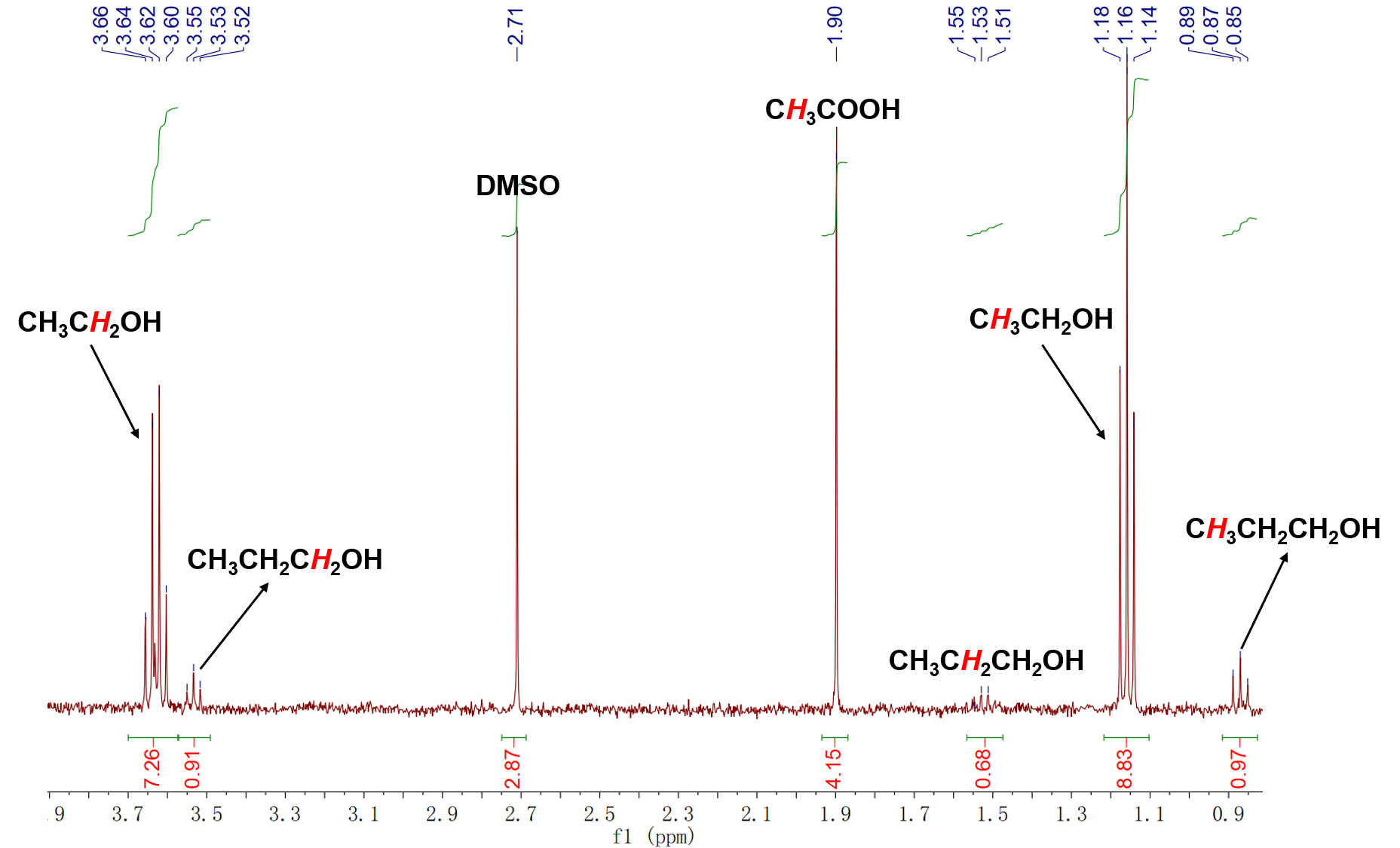


**Figure S9.** ^1^H NMR spectrum (400 M, 10% D_2_O + 90% H_2_O, water suppression, 8 ppm DMSO) of liquid products acquired from Cu NW/Me-GDY for CO_2_RR at –1.2 V vs. RHE in 1 M KOH.


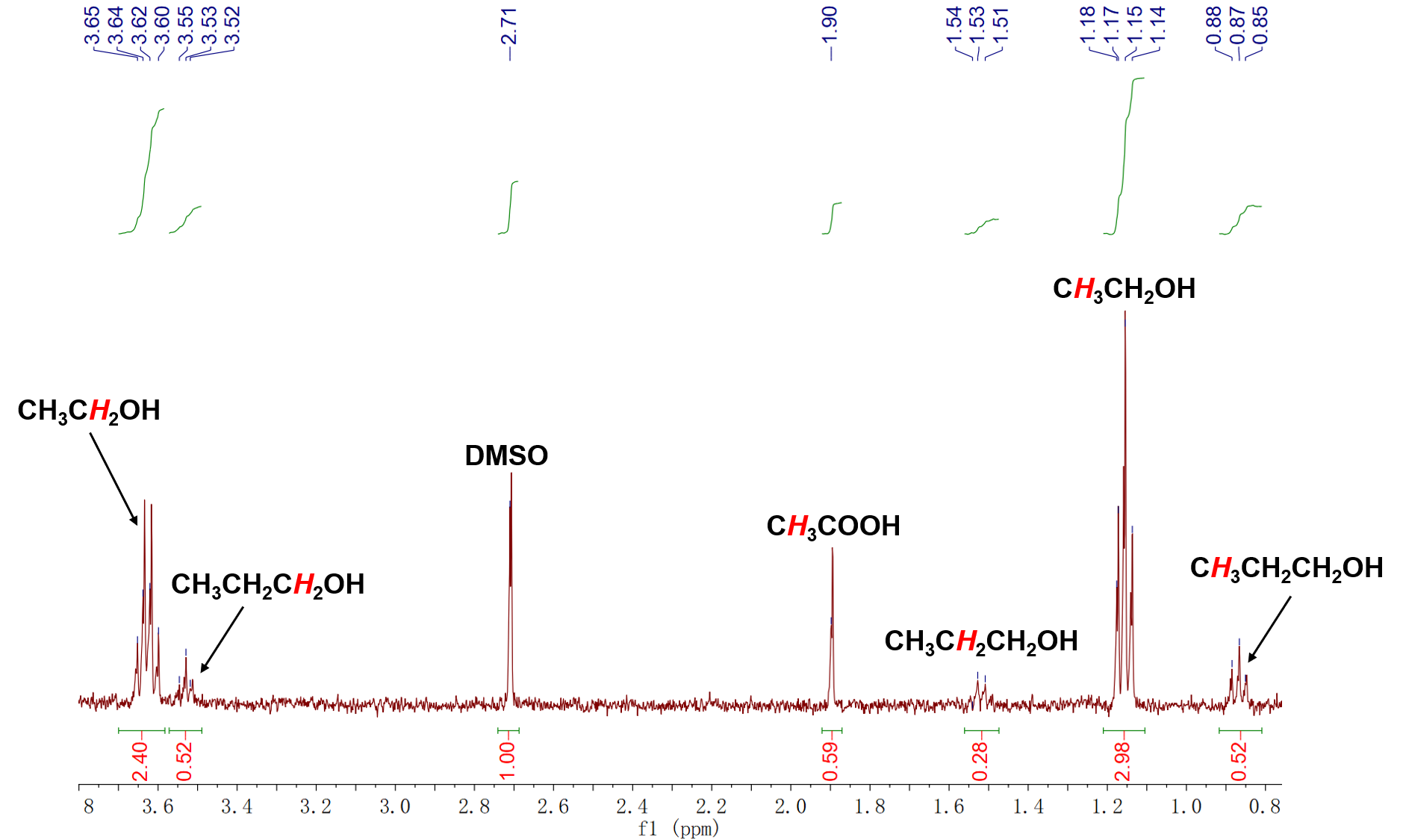


**Figure S10.** ^1^H NMR spectrum (400 M, 10% D_2_O + 90% H_2_O, water suppression, 8 ppm DMSO) of liquid products acquired from Cu NW/F-GDY for CO_2_RR at –1.2 V vs. RHE in 1 M KOH.


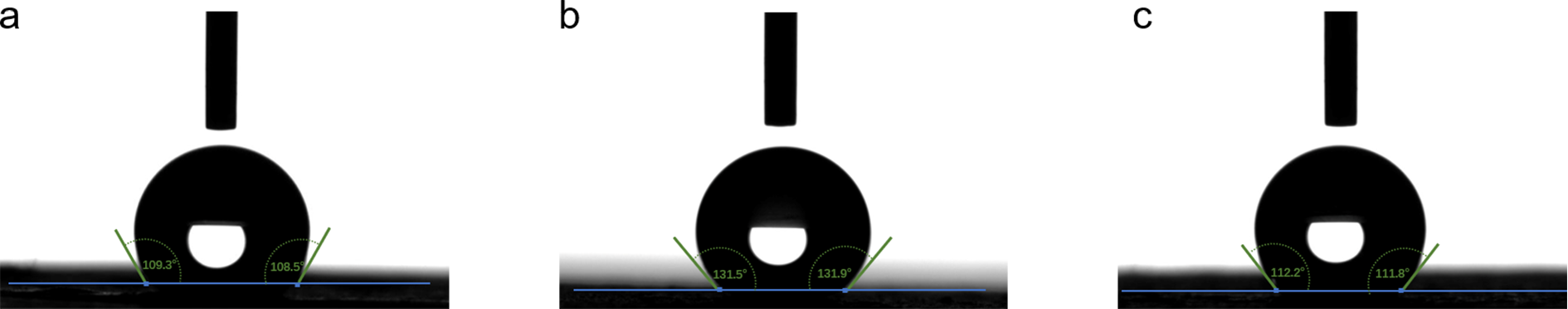


**Figure S11.** Contact angle measurements of (a) Cu NW, (b) F-GDY/Cu NW and (c) Me-GDY/Cu NW deposited on the GDL.


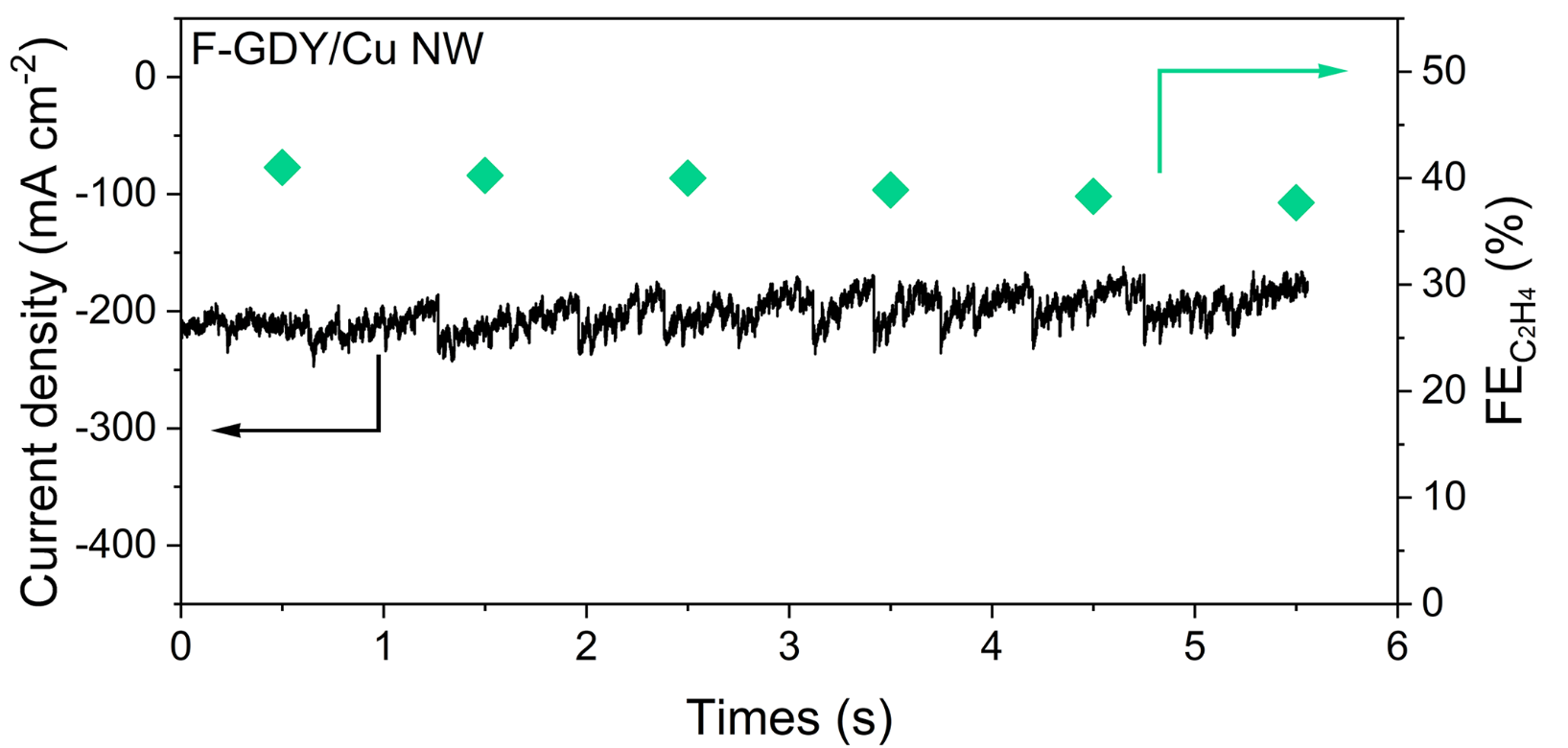


**Figure S12.** Stability test of F-GDY/Cu NW at –1.1 V *vs.* RHE.


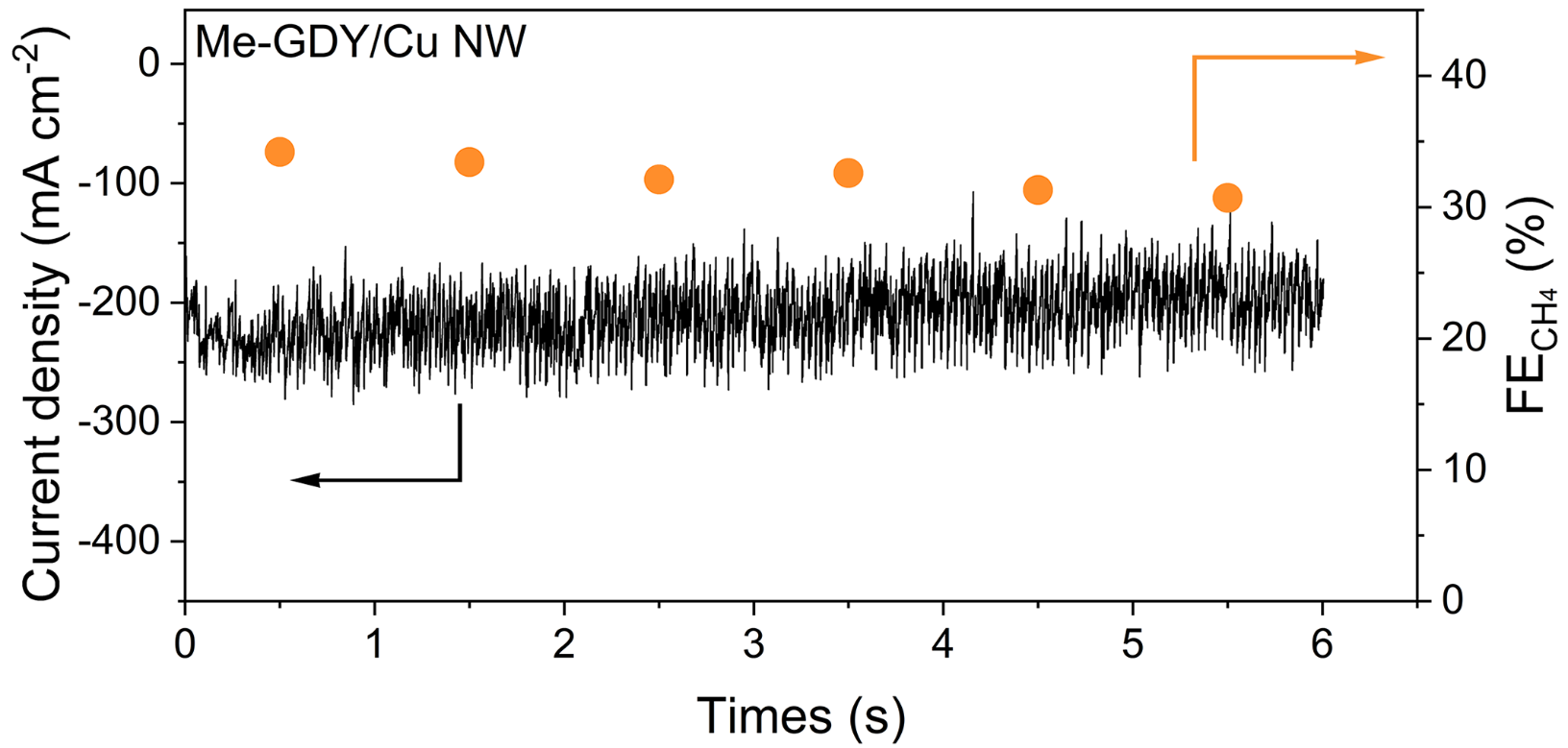


**Figure S13.** Stability test of Me-GDY/Cu NW at –1.1 V *vs.* RHE.


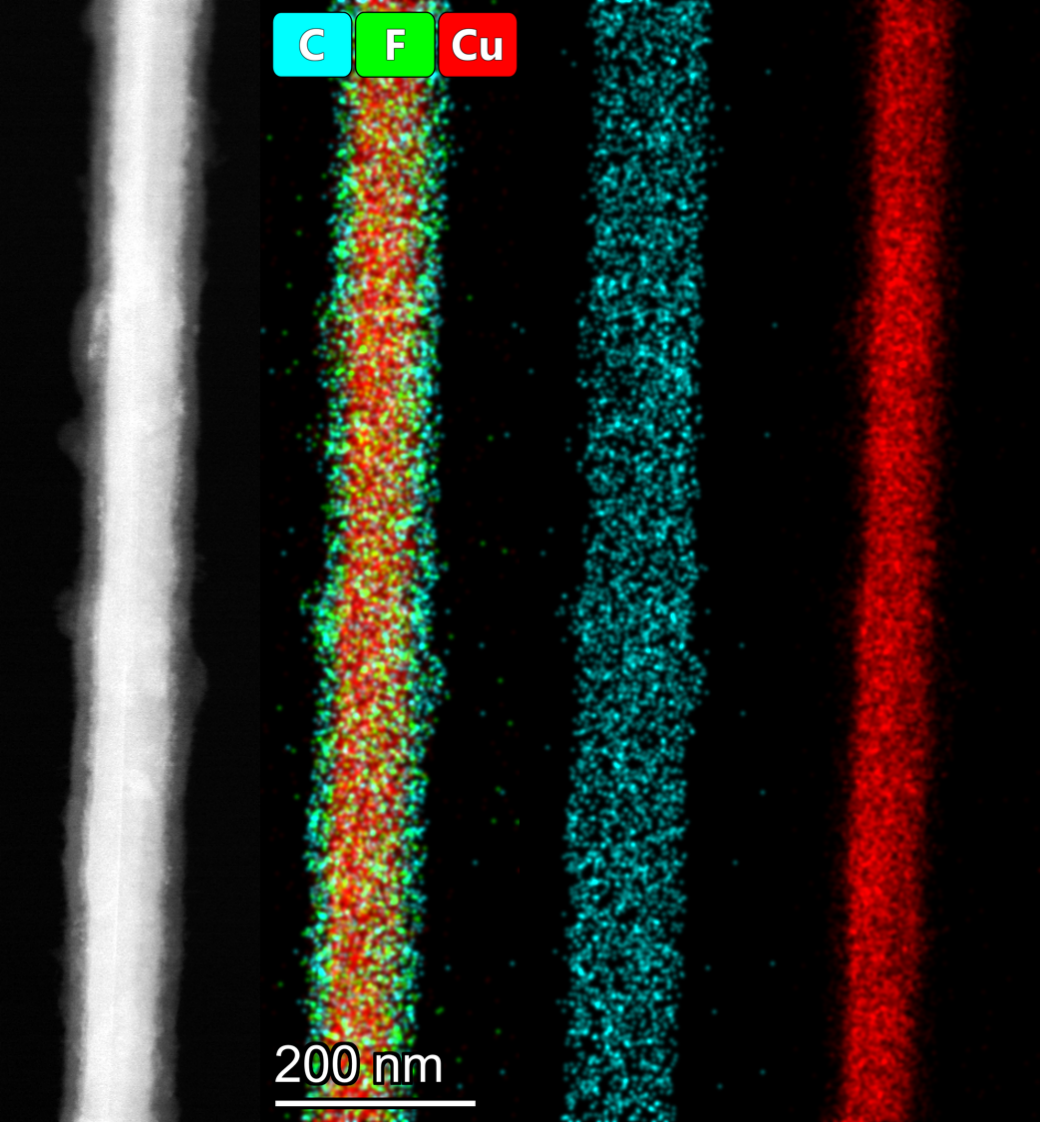


**Figure S14.** HAADF and correlated EDS mapping images of F-GDY/Cu NW after electrolysis.


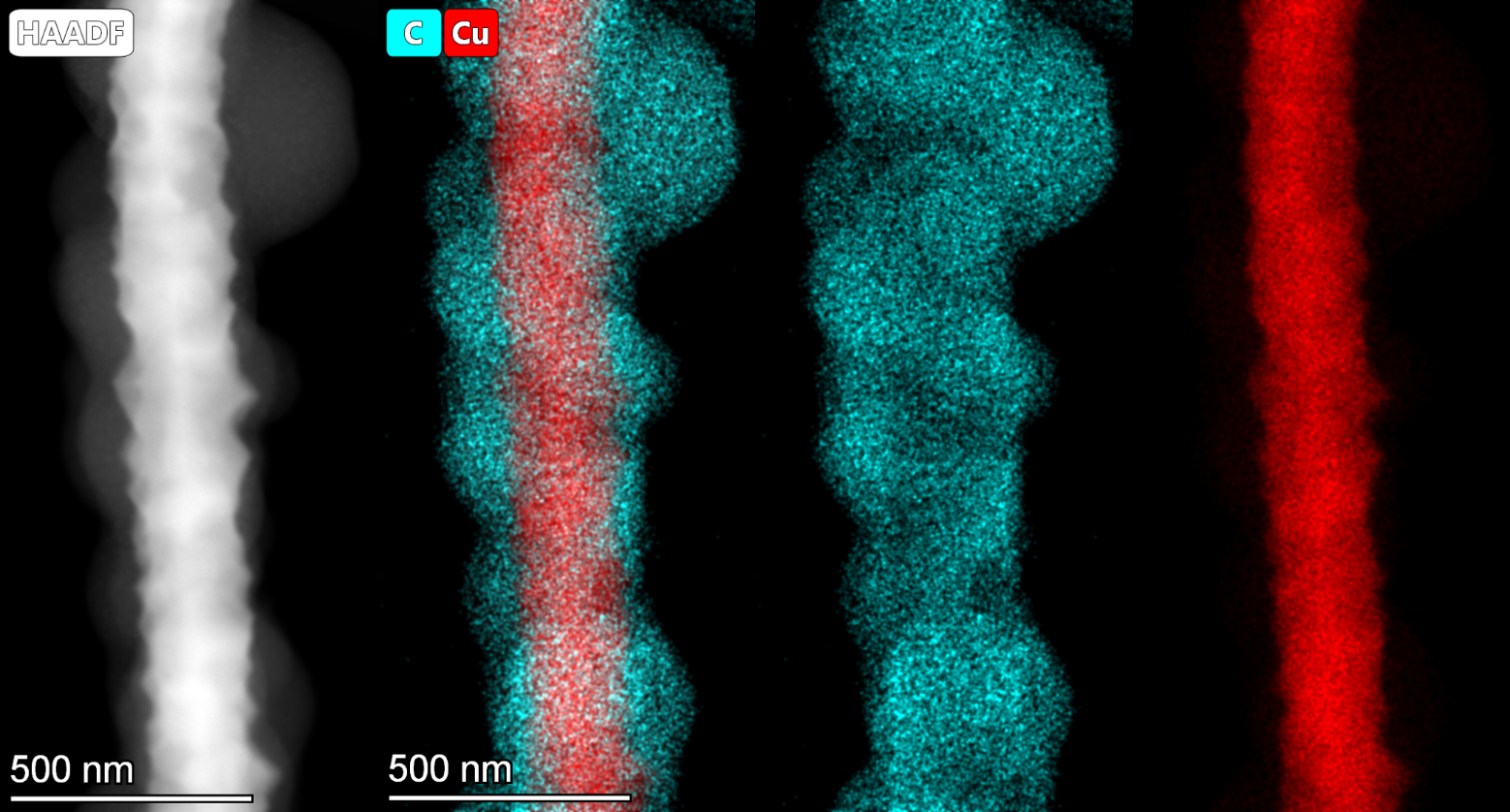


**Figure S15.** HAADF and correlated EDS mapping images of Me-GDY/Cu NW after electrolysis.


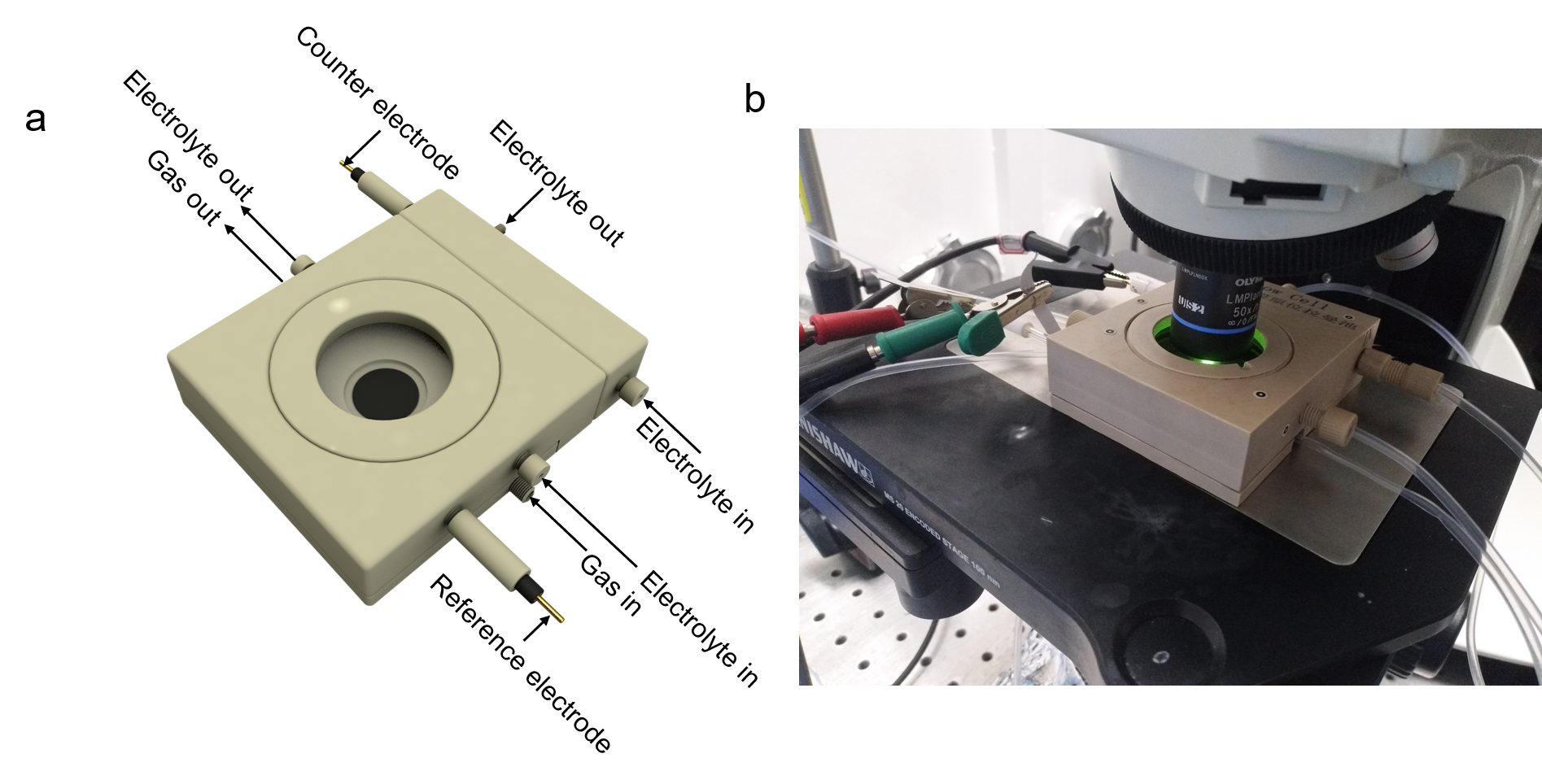


**Figure S16.** (a) Schematic of the in situ electrolyzer for Raman test. (b) Photograph of the corresponding set-up.

**Reference**

1. Jung SM, Preston DJ, Jung HY *et al.* Porous Cu nanowire aerosponges from one-step assembly and their applications in heat dissipation. *Adv Mater* 2016; **28**: 1413-1419.

2. Ferreira RB, Figueroa JM, Fagnani DE et al. Benzotrifuran (BTFuran): A building block for π-conjugated systems. *Chem Commun* 2017; **53:** 9590-9593.

3. Chen Z, Weseliński ŁJ, Adil K et al. Applying the power of reticular chemistry to finding the missing alb-MOF platform based on the (6, 12)-coordinated edge-transitive net. *J Am Chem Soc* 2017; **139:** 3265-3274.

4. Hennrich G, Lynch VM, Anslyn EV. Novel C_3_‐symmetric molecular scaffolds with potential facial differentiation. *Chem Eur J* 2002; **8:** 2274-2278.

5. Kresse G, Furthmuller J. Efficiency of ab-initio total energy calculations for metals and semiconductors using a plane-wave basis set. *Comp Mater Sci* 1996; **6**: 15-50.

6. Hammer B, Hansen LB, Norskov JK. Improved adsorption energetics within density-functional theory using revised perdew-burke-ernzerhof functionals. *Phys Rev B* 1999; **59**: 7413-7421.

7. Kresse G, Joubert D. From ultrasoft pseudopotentials to the projector augmented-wave method. *Phys Rev B* 1999; **59**: 1758-1775.

8. Henkelman G, Uberuaga BP, Jónsson H. A climbing image nudged elastic band method for finding saddle points and minimum energy paths. *J Chem Phys* 2000; **113**: 9901-9904.

9. Smidstrup S, Pedersen A, Stokbro K *et al.* Improved initial guess for minimum energy path calculations. *J Chem Phys* 2014; **140**: 214106.

10. Yu M, Trinkle DR. Accurate and efficient algorithm for bader charge integration. *J Chem Phys* 2011; **134**: 064111.
